# Functions of the Auditory Forebrain for Song Memory and Preference Behavior in Female Zebra Finches

**DOI:** 10.1523/ENEURO.0164-26.2026

**Published:** 2026-06-24

**Authors:** Yung-Chieh Liu (劉 永傑), Yuichi Morohashi (諸橋 雄一), Yoko Yazaki-Sugiyama (杉山(矢崎) 陽子)

**Affiliations:** Neuronal Mechanism for Critical Period Unit, OIST Graduate University, Kunigami, Okinawa 904-0495, Japan

**Keywords:** auditory, experience dependent, songbird, song preference

## Abstract

Individual male zebra finches develop a unique song, which females listen to and use to choose their mate. Both male and female zebra finches show preferences for songs from familiar males, such as their tutor, father, or mate. Lesioning of the caudomedial nidopallium (NCM), which is analogous to the mammalian higher auditory cortex, diminishes the recognition of previously experienced songs. Electrophysiological experiments showed that NCM neurons exhibit different habituation rates for repeated stimuli with tutor's song (TS) and unfamiliar song in adult males, and subsets of NCM neurons respond exclusively to TS in male juveniles. Those collectively suggest NCM is involved in song memories for various behaviors. However, whether NCM is involved in auditory memory for song preference in adult females has not been clarified. Here, we examined whether NCM is involved in preference for father's song (FS) and how auditory experiences shape NCM neuronal responses. Inducing cell death in NCM FS-responsive neurons reduced the number of hops particularly toward FS in adult females. In contrast, only a small proportion of FS-selective neurons was found in NCM and that proportion was not different between normally reared females and females reared without exposure to FS. Decoder-based modeling indicated that reduced fractions of FS-responsive neurons in the NCM neuronal ensembles results in reduced decoding accuracy specifically to FS. Taken together, our results suggest that NCM neurons of adult female zebra finches are necessary for song discrimination in FS preference behavior, while they do not store FS memories as selective responses.

## Significance Statement

Recognizing vocal signatures of familiar individuals and vocal learning from tutors require forming memories from auditory experiences. Neurons are thought to form a memory when exhibiting selective activity toward specific stimuli. We discovered that ablating father's songs (FS)-responsive neurons in the caudomedial nidopallium (NCM) tended to reduce preference to FS in adult female zebra finches. However, we also found that only a small fraction of neurons in female NCM were selectively responsive to FS and that the extent of FS selectivity was not different between females raised with or without FS exposure. These results contrast with previous findings in juvenile male zebra finches and suggest sex and age differences in the sensitivity and memory forms of NCM neurons.

## Introduction

Male zebra finches produce individually unique songs to attract females during courtship (Zann, 1996), and female zebra finches hear and interpret songs for mate selection and individual recognition (ten Cate and Mug, 1984; Brazas and Shimizu, 2002; Riebel, 2009; Elie and Theunissen, 2018; Barr et al., 2021; Wang et al., 2022). Both male and female zebra finches have been reported to show preference to familiar songs, such as songs of a father, tutor, or mate, over songs of unfamiliar males (Miller, 1979a,b; Clayton, 1988; Riebel et al., 2002; Fujii et al., 2021; Wei et al., 2022). Additionally, they can remember songs of a specific singer from a pool of songs, recognizing an average of >40 singers, thereby demonstrating their discriminability of individual signatures embedded in songs (Elie and Theunissen, 2018; Yu et al., 2020). These suggest that both male and female zebra finches memorize songs they hear. However, neither the neuronal mechanisms of song memorization nor those of song recognition for preference have been fully elucidated, particularly in females.

The auditory pathway is mostly shared between males and females (Fortune and Margoliash, 1995; Bolhuis and Gahr, 2006; Bolhuis and Moorman, 2015). The caudomedial nidopallium (NCM), a brain area analogous to the mammalian higher auditory cortex (Vates et al., 1996; Jarvis et al., 2005; Jarvis, 2019), has been reported to be involved in song recognition and preference behavior both in males and females (Mello et al., 1992; Mello and Clayton, 1994; Chew et al., 1996; Bolhuis et al., 2000; Stripling et al., 2001; Bailey et al., 2002; Terpstra et al., 2004, 2006; London and Clayton, 2010; Yoder et al., 2015; Diez et al., 2019; Woolley and Woolley, 2020). Inactivation of NCM neurons in females impairs their bias toward normal male songs over altered songs (Tomaszycki and Blaine, 2014), suggesting the involvement of NCM in conspecific song recognition. Furthermore, bilateral NCM lesions disrupt their capacity to discriminate individuals based on songs or calls in both adult males and females (Yu et al., 2023). A recent study revealed that a higher proportion of NCM neurons express pS6, a marker of neuronal activity and plasticity, in females listening to their mate's song than in those listening to a neighbor's song (Catalano and Woolley, 2025), suggesting that the NCM is also involved in song memory. Various studies have suggested song memories form in the NCM with electrophysiological experiments as well. NCM neurons show different habituation rates and sensitivity to learned tutor's songs (TS) in adult males compared with songs of unfamiliar male birds (Phan et al., 2006; Miller-Sims and Bottjer, 2014; Moore and Woolley, 2019). With electrophysiological recording in awake freely behaving condition, we reported a fraction of NCM neurons acquire highly selective auditory responsiveness to a TS by learning from it in male juveniles (Katic et al., 2022). While the functions of song memory storage and song recognition in NCM of adult females have been suggested, how song memories are represented in the female NCM is largely unknown.

Here, we investigated whether NCM is involved in father's song (FS) preference and the neuronal mechanism for FS memory in female zebra finches. We found that inducing cell death in the NCM FS-responsive neurons tended to reduce FS preference in adult females. In contrast, the proportions of FS-selective neurons in the NCM did not differ between females reared with or without exposure to FS, whereas response selectivity to songs, but not to song elements, was altered by their developmental FS experiences. The analysis with decoder-based modeling trained with responses of NCM neuronal ensembles to various song stimuli showed reduced accuracy of FS detection with smaller fractions of FS-responsive neurons. Our results suggest that NCM neurons of adult females do not store FS memories in a form of selective activities to it, unlike in male juveniles where TS-selective neurons are found. Rather, our results suggest that NCM neurons detect various songs as neuronal ensembles and are involved in song preference behavior.

## Materials and Methods

### Animals

All experiments were approved by the Committee for Care and Use of Animals at the Okinawa Institute of Science and Technology Graduate University under the guidance of the AAALAC.

Thirty adult female zebra finches (*Taeniopygia guttata*), hatched and raised in our colony, were used in this study (Extended Data Fig. 1-3). To guarantee sufficient exposure to the FS, 23 females were raised in a breeding cage together with their parents and siblings until at least 45 d posthatch (dph; median 73 dph, range 45–122 dph) and were moved to a cage with only female birds. When they reached adulthood (median 158 dph, range 122–502 dph), they were confirmed to show a robust preference for FS over an unfamiliar song (UF) in the song preference test (see details below, Preference test) and then subjected to neuronal ablation experiments (*N* = 10; median 147 dph; range 129–312 dph) or electrophysiological recordings (*N* = 13; median 202 dph; range 132–632 dph). Another subset of seven females was separated from their father at 10 dph and from their male siblings at 30 dph and isolated with their mother in a sound attenuation chamber (Muromachi Kikai) to prevent exposure to zebra finch songs until they were subjected to the electrophysiological experiments at adulthood (median 131 dph, range 123–201 dph). For the isolated females, the song preference test was not performed before the electrophysiological recordings to ensure no prior song experiences. All birds were maintained under a 14/10 h light/dark cycle and provided with food and water *ad libitum*.

### Song recording and song stimuli

Fathers of experimental birds were housed individually in a sound attenuation chamber that was equipped with a lavalier microphone (C417 PP, AKG Acoustics) connected to a computer via an audio interface (Fast Track Ultra 8R, M-AUDIO), and their emitted songs were recorded with a 32 kHz sampling rate using the Avisoft-Recorder software (Avisoft Bioacoustics). The song of one male adult that died >4 years ago and to which the experimental female birds had never been exposed was selected from our song library and used as UF. FS and UF were high-pass filtered at 0.4 kHz and edited in Avisoft SASLab Pro (Avisoft Bioacoustics) and Audacity (Muse Group). For the preference test, three song files consisting of two or three song motifs with introductory notes (∼2.0 s long) were created for each male bird. Hopping on the perch triggered playback of one of the three files randomly. For song playbacks to elicit auditory responses in NCM neurons in the females with viral injections, three song files were created, each of which included six or seven FS motifs with introductory notes (∼5.0 s long). For examining neuronal responses, the “song stimulus” and “song element stimulus” series were created. The song stimulus series contained ∼2-s-long clips of white noise, male and female distance calls, a Bengalese finch song, four unfamiliar zebra finch songs, the male sibling's songs, and the FS (Fig. 2*B*). All zebra finch songs in the song stimulus series contained introductory notes and 2–3 motifs. All the songs used in this study were undirected songs. The song element stimulus series consisted of 10 single elements clipped from different zebra finch songs for each of the eight types (80 elements in total) categorized in our previous study (Cheng and Yazaki-Sugiyama, 2025), to which the females had never been exposed (Fig. 3*A*). The order of Types 1–8 represented the high- to low-frequency occurrence in the zebra finch songs of our colony. The elements, Types 1, 2, and 3, resembled element types introductory, male distance call, and stack categorized by Zann (1993), whereas Type 6 element resembled female distance call (Elie and Theunissen, 2016). Type 4 included a noise-like component which sweeps down in pitch (∼60 ms, ∼2,800 Hz). Type 5 was a short and high-pitch note (∼20 ms, ∼4,800 Hz). Types 7 and 8 were short high-pitch stacks without and with down-sweep (∼40 ms, ∼3,200 Hz; ∼40 ms, ∼2,200 Hz), respectively. All stimuli were normalized to a volume of 70 dB in all experiments with custom MatLab (MATLAB R2019a, MathWorks) or Python (Python version 3.8, Python Software Foundation) code.

### Preference test

We used a two-choice operant conditioning paradigm, the perch-hop assay (Braaten and Reynolds, 1999), to assess the song preference of female zebra finches. A speaker was equipped at each side of a cage (width × depth × height = 40 × 30 × 40 cm), which was placed in a sound attenuation chamber (Muromachi Kikai). An experimental perch was mounted in front of each speaker. To detect the bird's hopping, each perch was equipped with an infrared optic switch (OPB100Z, TT Electronics) connected to a computer via a USB digital I/O device (USB-6501, National Instruments). Song playback from the adjacent speaker through a digital amplifier (PA3, Topping Audio) was triggered when a bird landed on the perch (Extended Data Fig. 1-1*A*). A neutral perch was placed in the center of the cage, in between the two experimental perches. The number of hops at each experimental perch was recorded using custom Python code. An experimental bird was introduced to the cage, and hopping three times on the experimental perches initiated the 20-min-long control trial in which hopping on the experimental perches did not trigger song playback (Extended Data Fig. 1-1*A*). After finishing six control trials, a song preference test was performed in which hopping on one experimental perch triggered a single playback of FS, whereas hopping on the opposite perch triggered a playback of UF. The same UF was used for all females to minimize variations in preference with songs. The birds must trigger ≥3 playbacks of each song to initiate a 20-min-long testing trial. In the testing trial, if a bird triggered <10 playbacks, the result of that trial was discarded due to the low confidence to reflect the song preference. We provided either 6 or 12 trials in a preference test, based on the motivation of the birds. All birds subjected to further experiments finished ≥6 testing trials in which they triggered >10 playbacks. A 10-min-long resetting period, in which hopping onto the experimental perches did not trigger a song playback, was appended after each testing trial. The side of the experimental perch assigned to FS was randomly selected and then to another side in the next trial after resetting period, and this cycle was repeated three times (6 trials) or six times (12 trials). All the control and testing trials were video recorded through a wide-angle camera (BSW200MBK, Buffalo) set inside the sound attenuation chamber. Food and water were provided during the perch-hop assay *ad libitum*.

### Song preference analysis

The song preference index (*I_p_*) was calculated by the following formula:

$$I_{p}=\frac{\mathrm{Ho}p_{\mathrm{FS}}-\mathrm{Ho}p_{\mathrm{UF}}}{\mathrm{Ho}p_{\mathrm{FS}}+\mathrm{Ho}p_{\mathrm{UF}}},$$

where $\mathrm{Ho}p_{\mathrm{FS}}$ and $\mathrm{Ho}p_{\mathrm{UF}}$ represent the numbers of hops triggering FS and UF, respectively. Positive $I_{p}$ indicates the preference for FS over UF in a trial. Birds that exhibited $I_{p}$ significantly greater than zero across all trials (*p* < 0.05, one-sample *t* test) were considered to prefer FS over UF.

### Surgery

For adeno-associated viral vector (AAV) injections or electrode implantations, birds were anesthetized by isoflurane inhalation (1.2–2.4%; 295 ml/min air flow, Univentor 400, Univentor) and fixed to a stereotaxic apparatus (Narishige), while their body temperature was maintained with a disposable warming pad held at ∼53°C (ONPAX, S.T. Corporation). The scalp was removed after the application of xylocaine jelly (2%, Sandoz Pharma K.K.), and then the skull and dura above the targeted areas were removed. In 10 birds, a cocktail of AAV(dj)-cFos-TetON-CaCaspase3 and AAV(dj)-cFos-TetON-dtA (Louder et al., 2024) was injected into the NCM according to the stereotaxic coordinates (18° head angle, 0.5 mm lateral and 0.5 mm anterior to the Y-sinus, 1.8 mm depth) through a glass pipette connected to the injector (Nanoject II, Drummond Scientific). Approximately 500 nl of viral solution was injected into each hemisphere over 7 min (18.4 nl each time, 46 nl/s, 15 s interval), and the glass pipette was removed from the brain after 15 min of stabilization. After the virus injections, the skull openings were sealed with silicone (Kwik-Cast, World Precision Instruments).

A 16-channel moveable bundle of tungsten electrodes (23 µm diameter, 3.5 mm guide cannula, 4 mm drive travel distance, 0.008″ silver ground wire; Innovative Neurophysiology) was implanted into 20 birds (seven were isolated birds), targeting the dorsomedial part of the NCM where we found more TS-selective neurons in male juveniles (Yanagihara and Yazaki-Sugiyama, 2016; Katic et al., 2022). The stereotaxic coordinates for electrode implantation were slightly more lateral and posterior than the one used for AAV injections to target the same area of NCM without hitting the field L with a thick electrode bundle (18° head angle, 0.60 mm lateral and 0.45 mm anterior to the *Y*-sinus, 1.1–1.3 mm depth; Fig. 2*A*; Extended Data Fig. 2-1*A*). A grounding wire was inserted under the skull over the anterior part of the contralateral hemisphere and fixed to the skull with dental cement (Super-Bond Bulk-mix kit, Sun Medical). Xylocaine and antibiotic gel (gentamicin sulfate, Fuji Pharma) were applied to the wound immediately, followed by twice a day for 3 d after the surgery.

### Neuronal ablation

**Figure 1.**
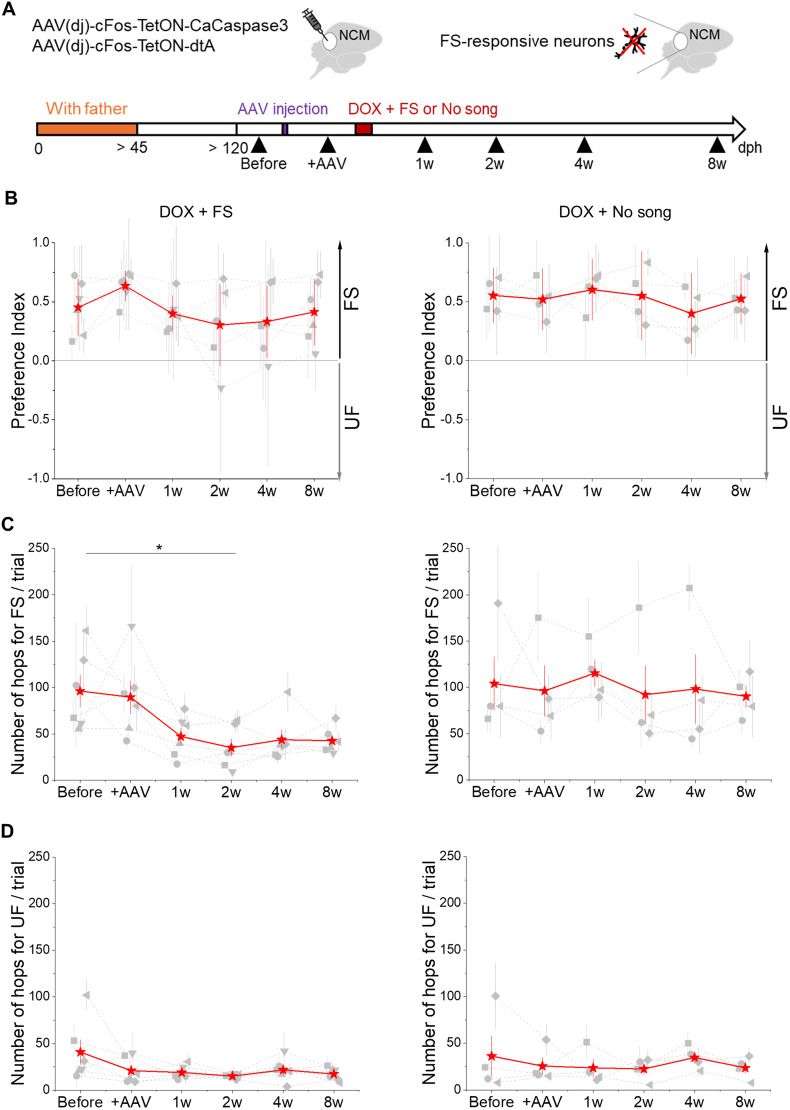
Preference strength to FS decreased after ablating FS-responsive NCM neurons in adult female zebra finches. ***A***, The timeline of neuronal ablation experiment. Arrowheads indicate the time points of song preference tests. dph, days posthatching. ***B–D***, The preference index for FS (***B***), the number of hops for FS (***C***), and that for UF (***D***) of the experimental birds (DOX + FS) and control birds (DOX + No song) before and after ablating FS-responsive neurons. Red and gray symbols denote the average across all individuals and the average across trials at each time point within each individual, respectively (6 experimental birds and 4 control birds). The different symbols denote each individual within a group (DOX + FS or DOX + No song). The number of hops for FS significantly decreased at 2 weeks after the neuronal ablation compared with before [*, *F*_(5,25)_ = 5.96; *p* = 0.001, one-way repeated–measures ANOVA; *t*_(5)_ = −6.20; *p* = 0.029, post hoc Bonferroni’s test between (2 w) 35.2 ± 23.0 (mean ± SD) hops and (Before) 96.2 ± 42.6 hops], whereas no changes were observed in the control birds at all time points (*F*_(5,15)_ = 0.17; *p* = 0.971, one-way repeated–measures ANOVA). Error bars indicate 95% confidence intervals in ***A*** and standard errors in ***C*** and ***D***; DOX, doxycycline; FS, father's song; UF, unfamiliar song. Refer to Extended Data Figure 1-3 for the list of female zebra finches used and Extended Data Figure 1-1 for additional information about the preference test and viral expression. Refer to Extended Data Figure 1-2 for the results of electrophysiological recordings in NCM before and after neuronal ablation.

The birds were kept in a sound attenuation chamber and tested for song preference again at two weeks after the injections. The birds were then provided with doxycycline (DOX; 0.4 mg/ml, 324385-1GM, Millipore) in the drinking water with 5% sucrose, starting in the evening before the song playback. FS playback (total ∼1 h) was provided in the morning of the next 2 d. The control birds were provided with DOX water and no song playback. The DOX water was removed on the morning of the second day of song playback. All birds were kept in sound attenuation chambers afterward and subjected to song preference tests at 1, 2, 4, and 8 weeks after the DOX water removal (Fig. 1*A*).

### Electrophysiological recording

After recovering from electrode implantation surgery (12–24 h), the birds underwent electrophysiological recordings. The headstage (HST/16o25-GEN2-18P-2GP-G1, Plexon) was connected to the electrode and then to the neural data acquisition system (OmniPlex, Plexon). Neuronal signals were amplified 1,000-fold, bandpass filtered at 0.5–9.0 kHz, and digitized at 40 kHz using the OmniPlex System (Plexon). At each recording site, neuronal activities were recorded after a 5–10 min adjustment period. During recording, song stimuli were amplified (TP21T-Amp Class T Mini Amplifier, Topping Audio) and played from the speaker by a custom-written LabView code (LabVIEW 2016 64-bit version, National Instruments). Neuronal responses to the song stimuli and the timing of song stimulation were recorded and saved on the PC. Each stimulus was presented 10 times in a pseudorandomized order, with 10 s intervals (or 5 s intervals for song elements stimulus series). After recording neuronal activities at one site, the electrodes were advanced 0.1–0.2 mm along the dorsal–ventral axis with confirmation of disappearance of neuronal activities and appearance of new units, and the new recordings were performed after 5–10 min adjusting time. The neuronal recordings were terminated once the electrode reached the ventral edge of NCM (∼2.8 mm depth). Neuronal recordings for each bird in a single experiment were completed within 2 d.

### Electrophysiological data analysis

We performed spike sorting with modified protocols from Cheng and Yazaki-Sugiyama (2025) using the Offline Sorter v3 software (Plexon). We auto-thresholded the spike activities at the threshold of six times the standard deviation from the mean amplitude and then identified putative single-unit spikes with visual inspection and the Find Units function of the software in each channel. In case a boundary between spike clusters in principal component analysis (PCA) space exhibited a truncation in its Gaussian-like distribution, a threshold was manually adjusted (not lower than three times the standard deviation from the mean amplitude) until a spike cluster appeared to be Gaussian-like distribution in the PCA space with visual inspection. The separation between each cluster was carefully secured, and the neuronal activities were further sorted into single units based on visual inspection (or discarded if a Gaussian-like cluster and good separation in PCA space cannot be achieved).

**Figure 2.**
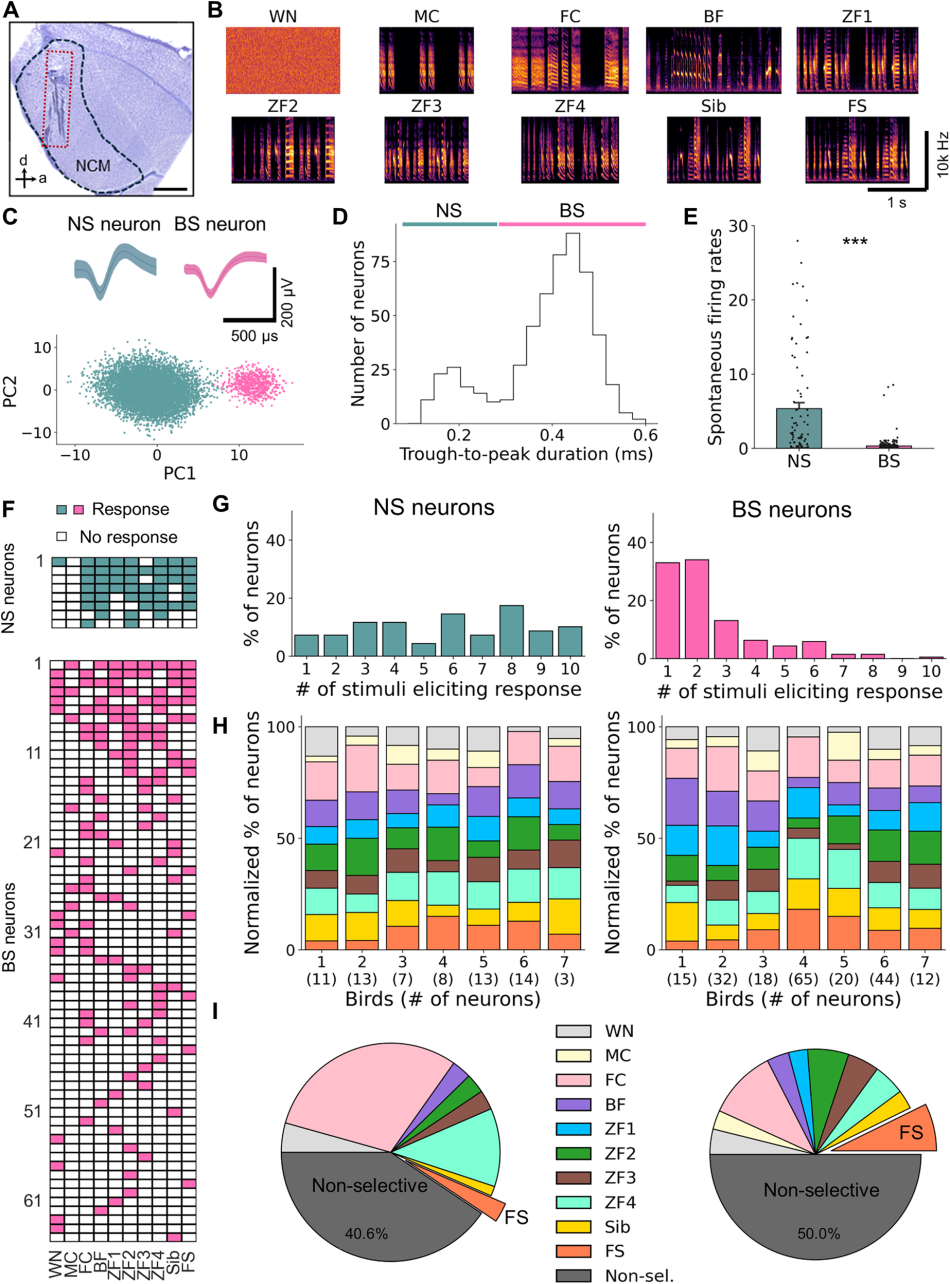
NCM neurons responded to a variety of zebra finch songs, but not selectively to FS. ***A***, Image of the Nissl-stained sagittal brain section showing the electrode implantation site. Black dashed line denotes the estimated outline of the NCM, and red dashed box highlights the traces of the electrodes. Scale bar, 500 µm; a, anterior; d, dorsal. ***B***, Sound spectrograms of 10 song stimuli in the song stimulus series, including white noise (WN), male calls (MC), female calls (FC), Bengalese finch song (BF), novel zebra finch songs (ZF1–ZF4), sibling's song (Sib), and FS. ***C***, Representative spike waveforms of NS and BS NCM neurons (top) and principal component (PC) analysis of those waveforms (bottom). ***D***, The histogram of trough-to-peak durations of NCM neurons recorded from adult females (*n* = 366 neurons; *N* = 7 birds). NS and BS neurons were divided with a duration of 0.3 ms [NS, 0.20 ± 0.04 (mean ± SD) ms; BS, 0.43 ± 0.06 ms]. ***E***, Average spontaneous FRs of NS and BS neurons recorded from adult females. Black dots denote data of individual neurons. FR of NS neurons is significantly higher than that of BS neurons (NS, 5.36 ± 6.94 spikes/s; *n* = 80; BS, 0.32 ± 0.84 spikes/s; *n* = 286; ***p = 2.11 × 10^−22^; *U* = 19,58;, *r* = 0.51, Mann–Whitney *U* test; error bars, standard errors). ***F***, Responses of individual NS (top) and BS (bottom) neurons to each song stimulus recorded from a representative normally reared adult female. Filled boxes denote significant responses (*p* < 0.05, Wilcoxon signed-rank test). ***G***, The percentage of NS (left) and BS (right) neurons regarding the number of song stimuli responded to in the song stimulus series. (NS, *n* = 69; BS, *n* = 206). NS neurons responded to a significantly greater number of song stimuli than BS neurons did (NS, 5.8 ± 2.8 stimuli; BS, 2.5 ± 1.7 stimuli; *U* = 11,860; *p* = 2.02 × 10^−17^; *r* = 0.50, Mann–Whitney *U* test). ***H***, Normalized percentage of NS (left) and BS (right) neurons responding to each stimulus in individual adult females. ***I***, Proportions of neurons selective for each song stimulus within all responding NS (left) and BS (right) neurons. The proportion of female-call-selective NS neurons is significantly greater than that of NS neurons selective for other stimuli except for ZF4 (WN, 4.3%; MC, 0.0%; FC, 30.4%; BF, 2.9%; ZF1, 0.0%; ZF2, 2.9%; ZF3, 2.9%; ZF4, 11.6%; Sib, 1.4%; FS, 2.9%; *χ*^2^_(9)_ = 94.10; *p* = 2.44 × 10^−16^, *χ*^2^ test; OR = 0.493; all *p* < 0.05 except for FC vs ZF4, post hoc Fisher's exact tests). The proportions of selective BS neurons are not different from each other (WN, 3.9%; MC, 2.9%; FC, 10.7%; BF, 3.4%; ZF1, 2.9%; ZF2, 6.3%; ZF3, 4.9%; ZF4, 4.9%; Sib, 2.9%; FS, 7.3%; *χ*^2^_(9)_ = 21.33; *p* = 0.011, *χ*^2^ test; all *p* > 0.05, post hoc Fisher's exact tests). Refer to Extended Data Figure 2-1 for the traces of electrophysiological recordings and the raster plots of representative neurons.

Narrow-spiking (NS) neurons and broad-spiking (BS) neurons were categorized based on trough-to-peak duration of their spikes (Fig. 2*C*,*D*; NS, ≤0.3 ms; BS, >0.3 ms). The sorted spike data were then analyzed using a custom Python code unless otherwise stated. Spontaneous firing rates (FRs) of a neuron were calculated as the mean FRs in the two-second time windows before each stimulus onset (Fig. 2*E*). Response strength $\left(\bar{\mathrm{RS}}\right)$ for each stimulus was calculated by the following formula:

$$\bar{\mathrm{RS}}=\bar{FR_{\mathrm{sti}}}-\;\bar{FR_{\mathrm{pre}}},$$

where $(\bar{FR_{\mathrm{sti}})}$ and $\;(\bar{FR_{\mathrm{pre}})}$ represent the mean FR during song playback (from the beginning to the end of song playback or 250 ms for song element stimuli) and prestimulus period (the same duration period with song stimuli just before each song stimulus) across 10 song repeats, respectively. In case the FRs during song playback $(FR_{\mathrm{sti}})$ were significantly greater than those during the prestimulus period $(FR_{\mathrm{pre}})$ across 10 song repeats (Wilcoxon signed-rank tests, *p* < 0.05), neurons were considered as responding to the stimulus (Fig. 2*F*–*H*).

**Figure 3.**
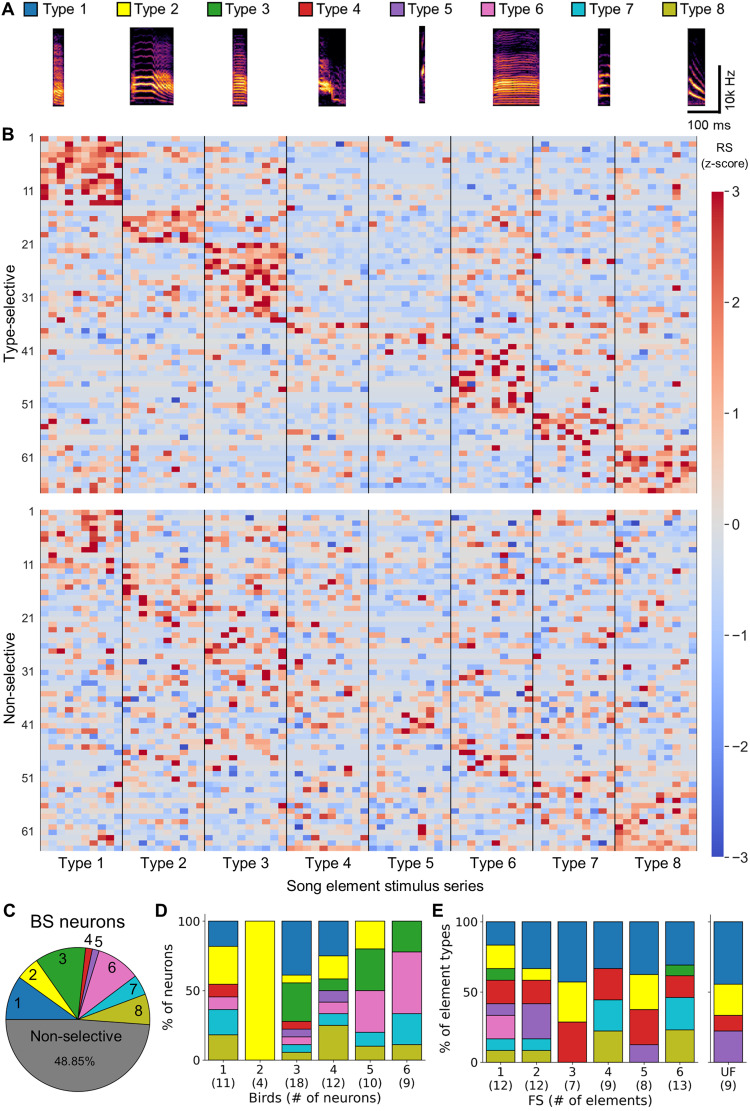
FS element-selective neuron was not a major population in NCM BS neurons. ***A***, Representative sound spectrograms of eight song element types in the song element stimulus series. ***B***, Heatmaps of the RS (*z*-score) of element type-selective (top) and nonselective (bottom) BS neurons to each of the 80 song elements in the song element stimulus series. ***C***, Proportions of selective neurons for each element type within the BS neurons that responded to the song element stimulus series (*n* = 131 neurons). The proportions of neurons selective for Types 4 and 5 are significantly less than that for Type 3 (Type 1, 9.9%; Type 2, 5.3%; Type 3, 11.5%; Type 4, 1.5%; Type 5, 1.5%; Type 6, 9.9%; Type 7, 4.6%; Type 8, 6.9%; *χ*^2^_(7)_ = 22.43; *p* = 0.002, *χ*^2^ test; *p* = 0.048 for Type 4 and Type 5 vs Type 3; OR = 0.65; *ϕ* = 0.05, post hoc Fisher's exact tests). ***D, E***, Percentages of BS neurons selectively responding to each element type in individual adult females (***D***) and percentages of the element types in their FS of corresponding adult females (***E***). The proportions of neurons that were selective to the element types in FS in one bird were not different from those selective to the element types in UF [FS, 64.0 ± 28.4% (mean ± SD); UF, 46.7 ± 31.3%; *W* = 1.0; *p* = 0.25; *r* = 0.81, Wilcoxon signed-rank test]. Refer to Extended Data Figure 3-1 for the element responsiveness of NCM NS neurons.

To examine the selectivity to a specific stimulus of neuronal responses, *d*′ values were computed for each stimulus pair using the following formula:

$$d^{\prime }=\frac{2(\bar{RS_{a}}-\bar{RS_{b}})}{\sqrt{\sigma_{a}^{2}+\sigma_{b}^{2}}},$$

where $\bar{RS_{a}}$ and $\bar{RS_{b}}$ represent RS for two stimuli (a and b), whereas $\sigma_{a}^{2}$ and $\sigma_{b}^{2}$ represent the variations of RS across 10 trials for each of the two stimuli. Neurons were considered selective to a specific stimulus if they exhibited *d*′ > 0.5 in comparison with all possible pairs with other stimuli, indicating an approximately twofold greater RS to a specific stimulus than to any other stimuli (Fig. 2*I*). For element type selectivity, RS was averaged across the song elements of the same type, and *d*′ was calculated for each pair of the eight types. Neurons with *d*′ > 0.5 for one type compared with all other types were classified as type-selective neurons (Fig. 3*B*–*D*).

### Statistical analysis

**Figure 4.**
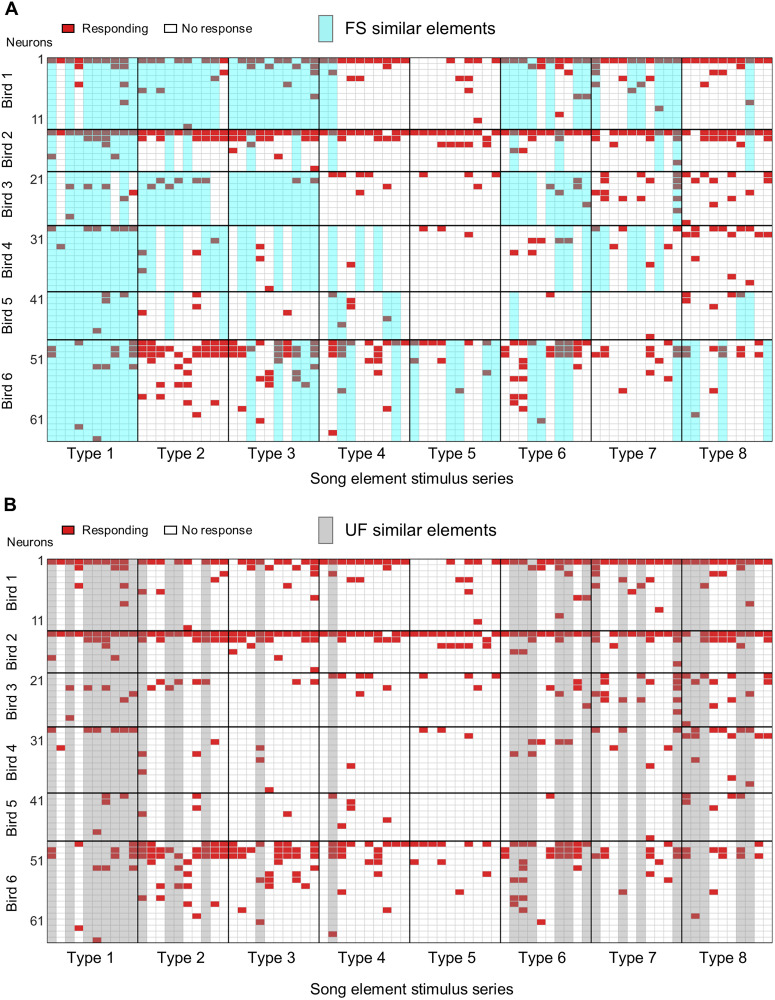
Responses of BS neurons are not biased to song elements similar to FS elements. ***A, B***, The responses to each song element stimulus within the song element stimulus series in individual nonselective BS neurons recorded from individual normally reared females. The highly similar (>80% similarity) elements with the FS (***A***) and UF (***B***) elements were highlighted in light blue (***A***) and gray (***B***), respectively. Red-filled boxes denote significant responses (*p* < 0.05, Wilcoxon signed-rank test). Nonselective BS neurons responded to comparable percentages of elements that are similar to the FS elements and those similar to the UF elements in each bird (all *p* > 0.05, Wilcoxon signed-rank tests).

**Figure 5.**
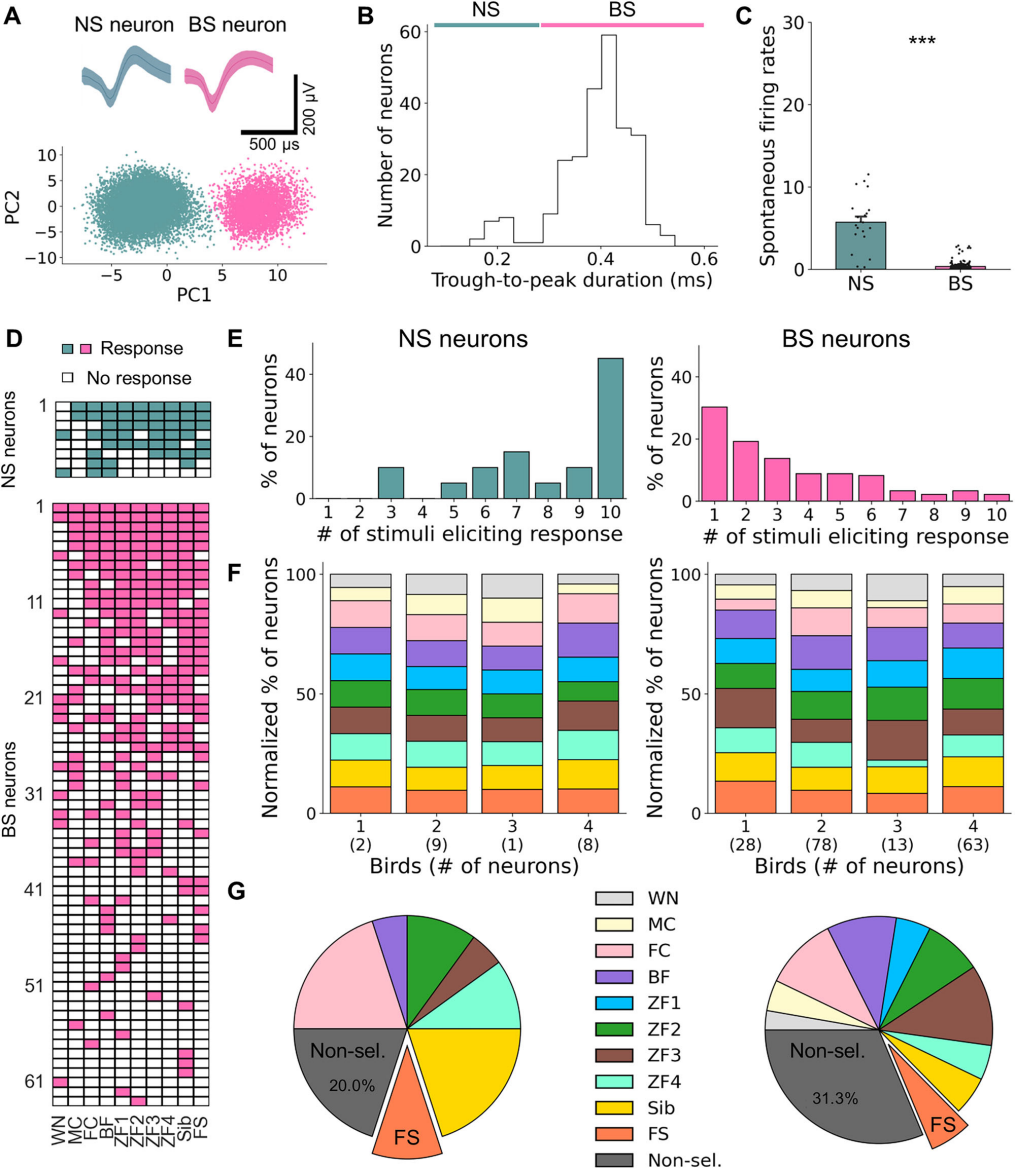
NCM neurons in isolated adult females responded to greater numbers of song stimuli. ***A***, Representative spike waveforms of NS and BS neurons (top) and the plot of their principal component (PC) analysis (bottom) recorded from isolated females. ***B***, Histogram of trough-to-peak durations of NCM neurons recorded from isolated females (*n* = 238 neurons; *N* = 4 birds). NS and BS neurons were divided with a duration of 0.3 ms [NS, 0.21 ± 0.04 (mean ± SD) ms; BS, 0.41 ± 0.05 ms]. ***C***, Average spontaneous FRs of NS and BS neurons in isolated females. Black dots denote the data of individual neurons. The FR of NS neurons is significantly higher than that of BS neurons (NS, 5.72 ± 3.15 spikes/s; *n* = 21; BS, 0.32 ± 0.49 spikes/s; *n* = 217; ***p = 1.58 × 10^−12^; *U* = 4,408; *r* = 0.46, Mann–Whitney *U* test; error bars indicate standard errors). ***D***, Responses of the NS (top) and BS (bottom) neurons to each song stimulus recorded from a representative isolated adult female. Filled boxes denote significant responses (*p* < 0.05, Wilcoxon signed-rank test). ***E***, The percentage of NS (left) and BS (right) neurons regarding the number of song stimuli responded within the song stimulus series (NS, *n* = 20; BS, *n* = 182). NS neurons responded to a significantly greater number of song stimuli than BS neurons did (NS, 8.0 ± 2.3 stimuli; BS, 3.3 ± 2.4 stimuli; *U* = 3,288; *p* = 1.92 × 10^−9^; *r* = 0.42, Mann–Whitney *U* test). ***F***, Normalized percentages of NS (left) and BS (right) neurons responded to each stimulus in individual isolated adult females. ***G***, Proportions of selective neurons to each song stimulus within all responding NS (left) and BS (right) neurons in isolated females. The proportions of selective neurons to each song stimulus are not significantly different from each other (NS, WN, 0.0%; MC, 0.0%; FC, 20.0%; BF, 5.0%; ZF1, 0.0%; ZF2, 10.0%; ZF3, 5.0%; ZF4, 10.0%; Sib, 20.0%; FS, 10.0%; *χ*^2^_(9)_ = 14.78; *p* = 0.097, *χ*^2^ test; BS, WN, 2.7%; MC, 4.4%; FC, 10.4%; BF, 9.9%; ZF1, 4.9%; ZF2, 8.2%; ZF3, 11.5%; ZF4, 4.9%; Sib, 5.5%; FS, 6.0%; *χ*^2^_(9)_ = 21.86; *p* = 0.009, *χ*^2^ test; all *p* > 0.05, post hoc Fisher's exact tests).

**Figure 6.**
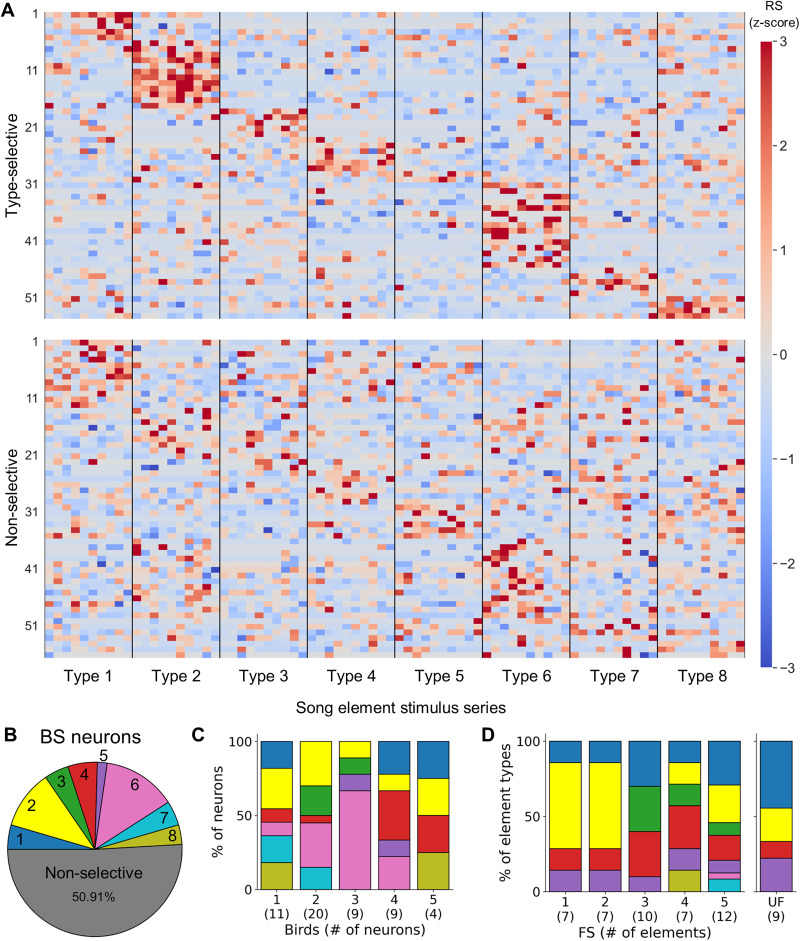
Selective responses to element types or specific song elements of the NCM BS neurons in isolated adult females. ***A***, Heatmaps of the RS (*z*-score) of type-selective (top) and nonselective (bottom) BS neurons to each of the 80 song elements in the song element stimulus series in isolated adult females. ***B***, Proportions of BS neurons selective for each element type in isolated females (*n* = 110 neurons). The proportion of Type 5 selective neurons is significantly smaller than that of Type 6 (Type 1, 4.5%; Type 2, 10.9%; Type 3, 4.5%; Type 4, 5.4%; Type 5, 1.8%; Type 6, 13.6%; Type 7, 4.5%; Type 8, 3.6%; *χ*^2^_(7)_ = 21.39; *p* = 0.003, *χ*^2^ test; *p* = 0.045 for Type 5 vs Type 6; OR = 1.26; *ϕ* = 0.02, post hoc Fisher's exact test). ***C, D***, Percentages of BS neurons selectively responding to each element type in individual isolated females (***C***) and percentages of element types in their genetic FS of each corresponding isolated female (***D***). The proportions of neurons that were selective to the element types in the genetic FS of each isolated bird were similar to those selective to the element types in UF [FS: 52.9 ± 21.8% (mean ± SD); UF, 52.9 ± 21.8%; *W* = 1.0; *p* = 1.0; *r* = 0.91, Wilcoxon signed-rank test].

All statistical analyses were performed using SciPy (Virtanen et al., 2020), except ANOVA and the post hoc tests for which Pingouin was used (Vallat, 2018) in Python. To assess the effects of neuronal ablation on the strength of FS preference (preference index, number of hops for FS and UF) at different timepoints in the neuronal ablation experiments, we used one-way repeated–measures ANOVA with post hoc pairwise comparison with Bonferroni’s correction for multiple comparisons (Fig. 1*B*–*D*). Greenhouse–Geisser correction to *p* values was applied when the sphericity assumption was violated in ANOVA analyses. The effects of the age removed from a breeding cage and of the age subjected to experiments on FS preference were checked by Spearman correlation (Extended Data Fig. 1-3). To compare properties (such as spontaneous FR, trough-to-peak duration, number of stimuli eliciting a response) between neural types or between bird groups with different rearing conditions (normally reared and isolated), Mann–Whitney *U* tests were performed (Figs. 2*D*–*G*, 4*B*–*E*). To compare the proportions of selective neurons for different song stimuli or different song element types, we performed *χ*^2^ tests and post hoc Fisher's exact tests with Bonferroni’s correction for multiple comparisons (Figs. 2*I*, 3*C*, 5*G*, 6*B*). Statistical analysis on the proportion of NS neurons was not performed due to the small sample size in individual birds (<10). To compare the proportions of selective BS neurons between normally reared and isolated birds, the proportion of single birds between the two groups was compared using the Mann–Whitney *U* test (Figs. 2*I*, 5*G*). To see if neurons in a given female were selective to the song element types existing in its FS, the proportions of BS neurons selectively responding to element types included in FS and those selectively responding to element types in UF in individual female birds were compared by using Wilcoxon signed-rank test (Figs. 3*D*,*E*, 6*C*,*D*).

### Analysis of neuronal responses to elements similar to FS elements

**Figure 7.**
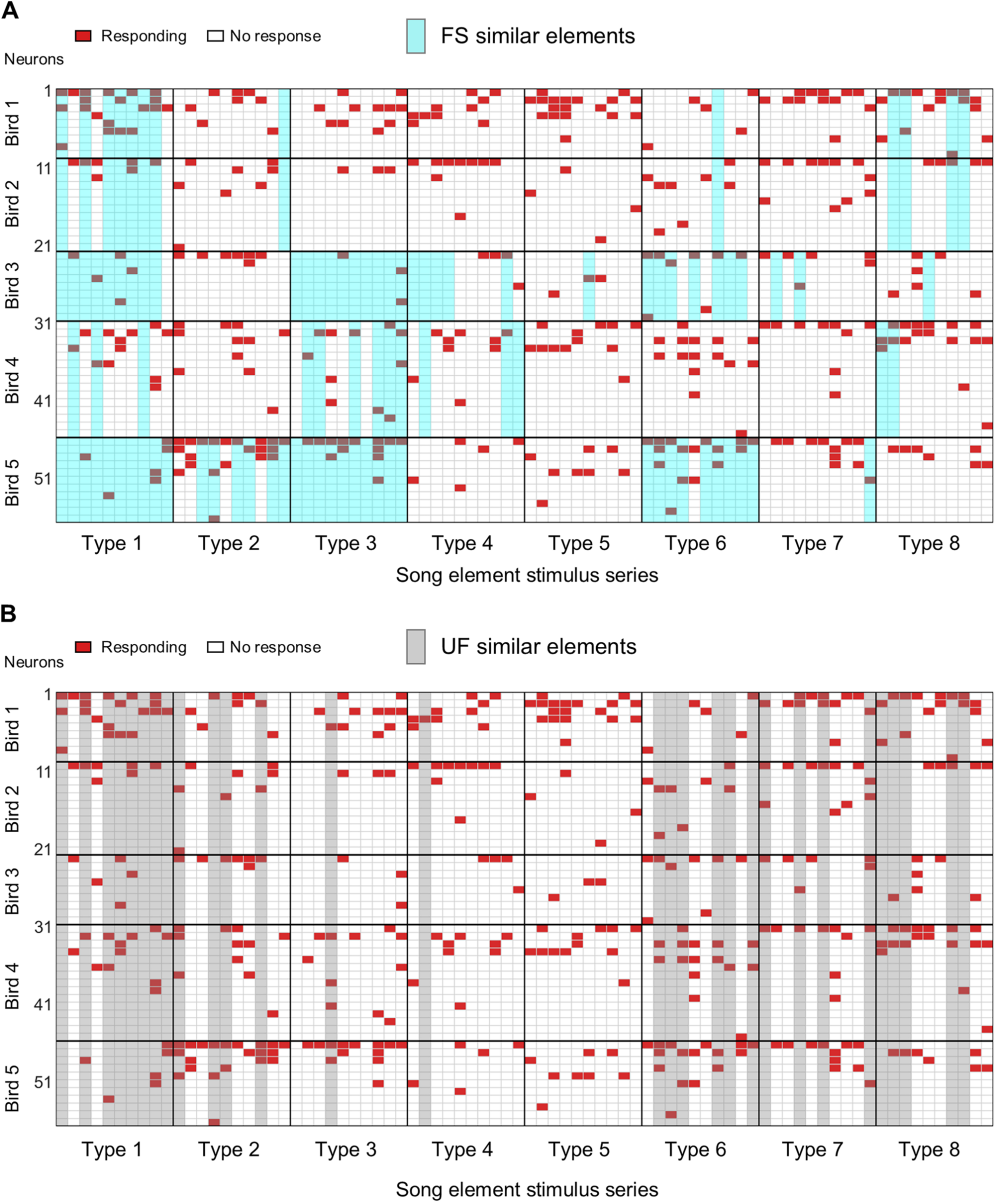
Responses of BS neurons are not biased to the elements similar to the FS elements in isolated adult females. ***A, B****,* Responses to each song element within the song element stimulus series in individual nonselective BS neurons recorded from individual isolated females. The highly similar (>80% similarity) elements with the FS (***A***) and UF (***B***) elements were highlighted in light blue (***A***) and gray (***B***), respectively. Red-filled boxes denote significant responses (*p* < 0.05, Wilcoxon signed-rank test). The percentages of elements that are similar to the elements of their genetic FS and those of the elements similar to the elements of UF within the song elements, to which nonselective BS neurons responded, were comparable in three isolated bird (*p* > 0.05, Wilcoxon signed-rank tests). In remaining two isolated birds, the percentages of elements that are similar to UF elements were higher than those of elements similar to FS within the elements to which nonselective BS neurons responded [Bird 1, UF 48.2 ± 41.3% (mean ± SD) FS 32.5 ± 30.4%; *W* = 0.0; *p* = 0.031; *r* = 0.89; Bird 2, UF 43.2 ± 24.2% FS 2.5 ± 5.5%; *W* = 0.0; *p* = 0.008; *r* = 0.88, Wilcoxon signed-rank tests].

We examined whether nonselective BS neurons preferentially responded to specific elements in the song element stimulus series which were acoustically similar to FS or UF elements. The similarities between each of the 80 elements in the song element stimulus series and the elements in FS (7–13 elements) or UF (9 elements) were calculated using Sound Analysis Pro 2011 (Tchernichovski et al., 2000), and >80% similarity was considered as highly similar (Figs. 4, 7). We tested whether the percentages of the elements that were highly similar to FS elements were significantly higher than those of the elements highly similar to UF elements within the elements individual neurons responded to in each female by using Wilcoxon signed-rank tests.

### Histology

The recording sites were confirmed by post hoc histological analysis. The birds were deeply anesthetized by intramuscular injection of ionol sodium (5 mg/ml, 0.01 ml/g of body weight) and transcardially perfused with ice-cold saline followed by 4% paraformaldehyde (PFA). Brains were dissected out, postfixed in 4% PFA overnight at 4°C, and then cryoprotected in 30% sucrose in phosphate-buffered saline (PBS) overnight at 4°C. Parasagittal sections (50 µm thickness) around the NCM were made using a freezing microtome (REM-710, Yamato Koki). The brain slices were washed with PBS (three times, 20 min each), mounted onto glass slides (MAS-01, Matsunami Glass), and dried at room temperature for 4–6 h. The brain sections were de-fatted in 95% ethanol at 60°C overnight, rehydrated with distilled water for 30 min, and then incubated with cresyl violet at 60°C (0.1%, 30 s) for Nissl staining. The sections were decolored in 70% ethanol with 0.1% acetic acid (40–60 s), underwent a dehydration series (80, 90, 95, 99.5%, absolute ethanol, and xylene), and were coverslipped with a multimount solution (FM22001, Matsunami Glass). All the brain sections around the recording site were imaged using the EVOS M7000 imaging system (Thermo Fisher Scientific) to estimate the recording sites. The electrode implantation sites were identified with tissue damage, and the electrode tracks were confirmed to be within the NCM (caudal from the edge of Field L) by visual inspection (Fig. 2*A*; Extended Data Fig. 2-1*A*).

### Estimating the AAV infection area within NCM

To estimate the area infected with AAVs, birds were injected with 500 or 200 nl of AAV(dj)-cFos-TetON-eGFP or AAV(dj)-cFos-TetON-mRFP [GFP, one bird, two hemispheres (500 nl); two birds, three hemispheres (200 nl); RFP, one bird, two hemispheres (500 nl); two birds, two hemispheres (200 nl)]. Two weeks after the injections of AAV, the birds were provided with DOX water together with FS playback and then were killed and perfused 5–7 d afterward. Their brains were collected and were cut into sagittal slices (50 µm thickness) using a freezing microtome. The brain slices were collected and washed with PBS (three times, 20 min each) and incubated with a primary antibody [rabbit anti-GFP (1:1,000; A11122, Invitrogen) or rat anti-RFP (1:1,000; 5F8, ChromoTek)] in 2.0% Triton X-100 in PBS at 4°C over two nights. The brain slices were then washed with PBS and further incubated with secondary antibody [goat anti-rabbit antibody conjugated with Alexa Fluor 488 (1:500; A11034, Invitrogen) or goat anti-rat antibody conjugated with Alexa Fluor 568 (1:500; A11077, Invitrogen)], together with 1 mg/ml DAPI solution (1:2,000; 19178-91, Nacalai Tesque) in 2.0% Triton X-100 in PBS at 4°C over two nights. Brain slices were again washed with PBS, mounted onto glass slides (MAS-01, Matsunami Glass), and coverslipped with mounting medium [Flouromount (K024-200, Dignostic BioSystems)]. For each hemisphere, a brain slice closest to the injection site, which was identified with the largest number of GFP- or RFP-positive neurons was found, was imaged as a *z*-stack image using a confocal microscope (A1R, Nikon) at 20× magnification (20×/0.75 NA, PlanApoVC, Nikon). The areas covered with GFP- or RFP-positive cell soma were determined in the *z*-stack maximum intensity projection image, and the contours of those areas were outlined and overlaid on a schematic of the brain slice containing NCM (Extended Data Fig. 1-1*B*).

### Electrophysiological recordings before and after neuronal ablation

To examine if the neuronal ablation occurred in the neurons which responded to the song stimuli (FS) we provided with DOX as expected, we recorded the neural responses to the song stimulus series before and after the neuronal ablation using AAV(dj)-cFos-TetON-CaCaspase3 and AAV(dj)-cFos-TetON-dtA in another subset of adult females (*N* = 4). These females were reared in the colony with their parents until at least 45 dph (median 62 dph, range 52–115 dph), like other normally reared females. We then confirmed their FS preference using the perch-hop assay after they became adults (median 243 dph, range 124–446 dph) and injected the AAVs bilaterally into their NCM (median 247 dph, range 130–458 dph). One week after the AAV injections, we unilaterally implanted an electrode into the NCM and recorded neural responses at 12–24 h after the electrode implantation (preablation). The electrode bundle was retracted back to the cannula after the recording to prevent degradation. Their FS preference was tested again 24–48 h after the recording. Two weeks after the AAV injections, the birds were provided with DOX water in the evening, then were exposed to FS playback for 1 h for 2 consecutive days in two birds (DOX + FS). In another two birds, DOX water was provided with no song playback (DOX + No song; Extended Data Fig. 1-2*A*). We then recorded their neural responses 2 weeks after DOX application (postablation). Those birds were kept in sound attenuation chambers after AAV injections, except during preference tests or neural recordings.

### Linear support vector machine decoder and decoder-based song prediction

**Figure 8.**
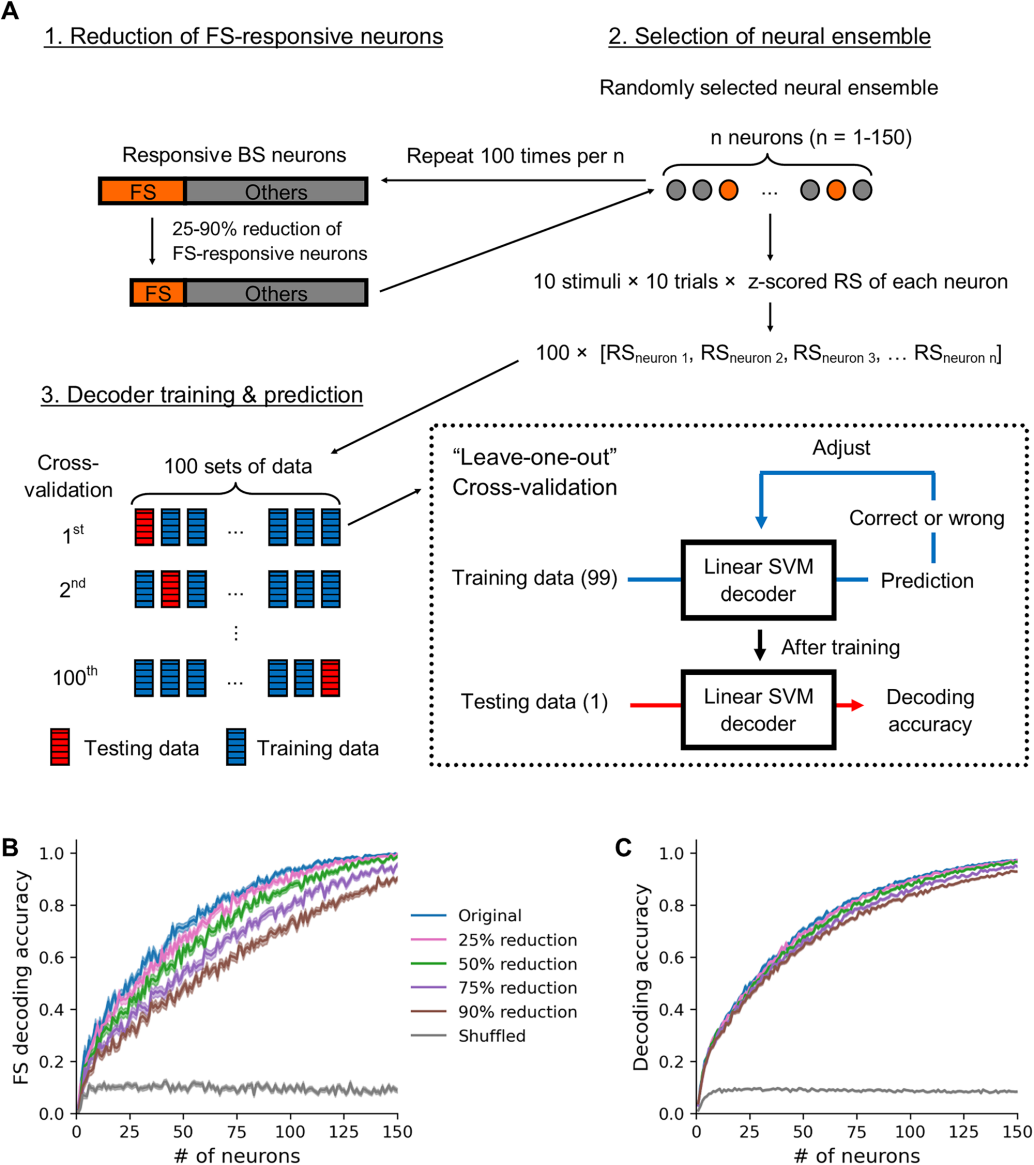
Decoding accuracy for FS decreased in the neural ensembles with smaller proportions of FS-responsive model neurons. ***A***, Pipeline of decoder analysis using the linear SVM decoder. The linear SVM decoder was trained by the RS of different numbers (ensemble size) of randomly selected NCM BS neurons recorded from normally reared females to each song stimulus of the song stimulus series. For each neuronal ensemble size, RS of BS neurons to 10 repeats of 10 stimuli in the song stimulus series (100 sets of stimulus and response) were used for training and testing. The decoder was trained by 99 sets of data and tested with remaining one if the correct song stimulus was predicted by the RS (leave-one-out), and these processes were repeated 100 times by making each set be used for testing and then calculated the percentage of correct predictions. The whole process, starting from random selection of neurons, was repeated 100 times for each ensemble size, and the average of the percentage of correct predictions was defined as decoding accuracy. The decoder was also trained by the RS of randomly selected neurons from the neuronal pools in which particular proportions (25, 50, 75, or 90%) of FS-responsive neurons were reduced. FS decoding accuracy was calculated as the average of the trials where FS was tested. ***B***, The decoding accuracy for FS for each neuronal ensemble size and with different proportions of FS-responsive model neurons. Decoding accuracy for FS decreased in the neural ensembles with reduced populations of FS-responsive model neurons (50, 75, and 90% reduction). ***C***, The decoding accuracy for all song stimuli for each neuronal ensemble size and with different proportions of FS-responsive model neurons. The decoding accuracy for all song stimuli was not different among the neuronal ensembles with reduced proportions of FS-responsive model neurons. The chance-level decoding accuracy was estimated by training the decoder and testing with the data in which combinations of RS and song stimuli were shuffled. Solid lines and shaded areas denote mean decoding accuracy and standard errors, respectively.

To estimate the discriminability of song stimuli in NCM neuronal ensembles, a linear support vector machine (SVM) decoder (SVC from Scikit-learn.svm with a linear kernel in Python) was used (Pedregosa et al., 2011). The decoder was trained using the *z*-scored RS to the stimuli in song stimulus series of 100 randomly sampled neurons from the 206 BS neurons recorded from normally reared adult females for each neuronal ensemble size (1–150). Neurons were uniformly sampled without repeated selection within each ensemble using a random generator function in Python (Numpy.random.Generator.choice). The decoder performance was evaluated using “leave-one-out” cross-validation method. The decoder was trained using 99 of 100 trials (10 stimuli × 10 trials) and tested in the remaining trial if a song stimulus was correctly predicted from the RS of the same ensemble. Training and testing were repeated 100 times by making each of 100 trials as a test, then the decoding accuracy was calculated as a percentage of correctly predicted trials. The decoding accuracy was averaged across 100 ensembles for each ensemble size. The chance-level decoding accuracy was calculated by training and testing the decoder using the data in which the song stimuli were randomly shuffled. For predicting with the reduced proportions of neurons responsive to FS, neurons were sampled from the neuronal pool where specific proportions (25, 50, 75, or 90%) of FS-responsive neurons were randomly removed (Fig. 8*A*), and the decoding accuracy was calculated in the same manner. The decoding accuracy for FS was calculated as the number of correctly predicted FS trials divided by the number of all FS trials tested.

## Results

### NCM neurons in adult female zebra finches are involved in FS preference

Previous studies have shown that inactivating NCM or caudal nidopallium in female songbirds decreases the strength of song preference or impairs vocal recognition of specific males (Tomaszycki and Blaine, 2014; Lawley et al., 2022; Yu et al., 2023). Here, we investigated whether NCM, especially the neurons responsive to FS playback, are involved in FS preference in adult female zebra finches. Adult zebra finch females (122–502 dph) were confirmed to show robust FS preference (*N* = 10; Fig. 1*B*–*D*, “Before”). Both the preference index and number of hops for FS were not correlated with the age when they were removed from their breeding cage and the age subjected to the experiment (Extended Data Fig. 1-3; preference index × cage removal age, *ρ* = −0.164; *p* = 0.182; preference index × test age, *ρ* = 0.053; *p* = 0.666; hops for FS × cage removal age, *ρ* = 0.195; *p* = 0.111; hops for FS × test age, *ρ* = −0.207; *p* = 0.091; Spearman correlation). Next, to test if the NCM FS-responsive neurons were necessary for female song preference to FS, cell apoptosis was induced in those neurons by injecting a cocktail of AAVs [AAV(dj)-cFos-TetON-CaCaspase3 and AAV(dj)-cFos-TetON-dtA] and presenting FS playback with DOX administration to adult females (*N* = 6; Fig. 1*A*). While neuronal ablation with viral expression of caspase and dtA has been used widely in mice and songbirds (Yang et al., 2013; Roberts et al., 2017; Sanchez-Valpuesta et al., 2019; Louder et al., 2024), identifying the proportion and sites of ablated neurons is technically limited. To estimate the area of neurons expressing CaCaspase3 or dtA, we injected the same amount of another viral vectors which induced GFP or RFP expressions under the same promoter in another subset of adult females with the same stereotaxic coordinates and confirmed that neurons across dorsomedial part of NCM expressed GFP or RFP, suggesting neurons in similar areas were ablated by expressing CaCaspase3 or dtA (Extended Data Fig. 1-1*B*). While preference strength for FS tended to decrease after the induction of cell death in the NCM neurons that responded to FS, including neurons both selectively and nonselectively responding to FS, the level of decrease did not reach to significance at any time points (*N* = 6; *F*_(5,25)_ = 2.43; *p* = 0.063, one-way repeated measures ANOVA; all *p* > 0.05, post hoc Bonferroni’s tests; Fig. 1*B*, left). These females hopped more for FS than UF across the time course of experiments (Fig. 1*C*,*D*, left); however, the number of hops for FS, but not for UF, was significantly reduced at 2 weeks after the ablation of FS-responsive neurons [FS, *F*_(5,25)_ = 5.96; *p* = 0.001, one-way repeated–measures ANOVA; *t*_(5)_ = −6.20; *p* = 0.029, post hoc Bonferroni’s test between (2 w) 35.2 ± 23.0 (mean ± SD) hops and (Before) 96.2 ± 42.6 hops; UF, *F*_(5,25)_ = 2.30; *p* = 0.075, one-way repeated–measures ANOVA; all *p* > 0.05, post hoc Bonferroni’s tests]. As the number of hops varied between individuals, we confirmed a statistically significant decrease with the ablation of FS-responsive neurons even without the data from one female that showed the most dramatic changes (*F*_(5,20)_ = 7.88; *p* = 0.0003, one-way repeated–measures ANOVA), while the post hoc pair comparison was no longer significant [*t*_(4)_ = −5.29; *p* = 0.092; post hoc Bonferroni test between (2 w) 40.4 ± 21.3 hops and (Before) 103.2 ± 43.7 hops]. In contrast, the control adult females that received DOX administration with no exposure to song playback, in which smaller amounts of random NCM neurons were expected to be ablated, consistently showed song preference to FS (*N* = 4; *F*_(5,15)_ = 0.82; *p* = 0.555, one-way repeated–measures ANOVA; Fig. 1*B*, right). They performed consistent numbers of hops both toward FS (Fig. 1*C*, right; *F*_(5,15)_ = 0.17; *p* = 0.971, one-way repeated–measures ANOVA) and UF (Fig. 1*D*, right; *F*_(5,15)_ = 0.38; *p* = 0.855, one-way repeated–measures ANOVA). Both the experimental and control birds continued to perform more hops during testing trials, where the hops triggered song playbacks, than during control trials, where the hops did not, suggesting the neuronal ablation had no effect on song hearing or motivation to hop (Extended Data Fig. 1-1*C*). These results suggest that NCM neurons activated by FS playback are necessary for adult females to show behavioral preference toward FS.

10.1523/ENEURO.0164-26.2026.f1-1 Figure 1-1 Adult female zebra finches consistently hop more during testing trials than during control trials. **A**: Schematic of experimental perch-hop assay setup used in this study (left) and the diagram of experiment timelines (right). Hopping on the two experimental perches triggered song playbacks during testing sessions, whereas it did not during control sessions. **B**: The timeline for visualizing FS-responsive neurons (top), a representative image of NCM slice of injection site in the adult female zebra finch injected with AAV(dj)-cFos-TetON-eGFP (bottom left), and a drawing of the brain slice including NCM (bottom right). The colored contours denote the areas the GFP-positive or RFP-positive somata were found in each hemisphere injected with 200 nL (blue) or 500 nL (red) of AAV. a, anterior; d, dorsal. Scale bar, 500 μm. **C**: Average number of hops across birds performed during control (black) and testing trials (red) before and after the virus injection and NCM neuronal ablation in experimental birds (DOX + FS; N = 6) and control birds (DOX + No song; N = 4). Error bars, standard errors. Download Figure 1-1, TIF file.

10.1523/ENEURO.0164-26.2026.f1-2 Figure 1-2 Proportion of FS-responsive neurons decreased after ablating FS-responsive neurons. A: Timeline of extracellular recordings before and after the neuronal ablation. Arrowheads indicate the time points of extracellular recordings. B: Responses of individual BS neurons to each song stimulus recorded from individual experimental (DOX + FS) and control female birds (DOX + No song) before (Pre-ablation) and after (Post-ablation) neuronal ablation. Filled boxes denote a significant response (p < 0.05, Wilcoxon signed-rank test), among which orange boxes denote responses to FS. C: Proportion of FS-responsive BS neurons within all responsive BS neurons before (Pre) and after (Post) neuronal ablation in single experimental (DOX + FS) and control (DOX + No song) females. The proportions of FS-responsive neurons decreased after neuronal ablation in experimental birds, but not in control birds. Download Figure 1-2, TIF file.

10.1523/ENEURO.0164-26.2026.f1-3 Figure 1-3 The list of female zebra finches used in this study showing the ages removed from their breeding cages, subjected to the first preference test, and subjected to AAV injections or electrophysiological recordings. Download Figure 1-3, DOCX file.

### NCM neurons in adult female zebra finches are not biased to FS

In male juveniles learning songs from a tutor, a subset of NCM neurons have been found to show highly selective responses to a TS after tutoring. Those TS-selective neurons are suggested to be neuronal TS memory traits as the juveniles, in which TS-selective neurons were inhibited to emerge, did not learn TS (Katic et al., 2022). As we found that the ablation of NCM FS-responsive neurons reduced the number of hops triggering FS, we next examined if distinct populations of neurons showing selective responses to FS are found in NCM in adult females that showed a strong FS preference (preference index significantly greater than zero, *p* < 0.05, one-sample *t* test) with single-unit extracellular recording. We chronically implanted a 16-channel movable electrode into NCM of normally reared adult females (Fig. 2*A*; Extended Data Fig. 2-1*A*), and neuronal responses to playbacks of the song stimulus series, which included FS, white noise, male and female calls, a heterospecific Bengalese finch song, and novel zebra finch songs, were recorded in freely moving conditions (Fig. 2*B*). We found two types of neurons, NS and BS neurons (NS, 80 neurons, 8–16 neurons/bird; BS, 286 neurons, 22–85 neurons/bird; seven birds), which were distinct in their spike shapes and FRs, as previously reported in male zebra finches (Fig. 2*C*,*E*; Extended Data Fig. 2-1*B*,*C*; Schneider and Woolley, 2013; Yanagihara and Yazaki-Sugiyama, 2016; Bottjer et al., 2019). Consistent with the previous finding in males, NS neurons exhibited significantly shorter trough-to-peak duration (Fig. 2*D*; NS, 0.20 ± 0.04 ms; BS, 0.43 ± 0.06 ms), so NS and BS were classified based on the difference in their trough-to-peak duration with a threshold of 0.3 ms as used in a previous study in adult males (Cheng and Yazaki-Sugiyama, 2025). NS neurons exhibited significantly higher spontaneous FRs than BS neurons (Fig. 2*E*; NS, 5.36 ± 6.94 spikes/s; BS, 0.32 ± 0.84 spikes/s; *U* = 19,585; *p* = 2.11 × 10^−22^; *r* = 0.51, Mann–Whitney *U* test). The majority of both NS and BS neurons (NS, 86.3%, 69/80; BS, 72.0%, 206/286) showed significant responses to at least one stimulus in the song stimulus series (*p* < 0.05, Wilcoxon signed-rank test). We found that the majority of BS neurons responded to only a small subset of stimuli in the series [68/206 neurons (33.0%) responded to one and 70/206 neurons (34.0%) responded to two of 10 stimuli; Fig. 2*G*, right], whereas all the song stimuli in the series were responded by the different combinations of BS neurons recorded in almost all the birds tested (6/7 birds; Fig. 2*F*, bottom, *H*, right). We found that BS neurons, even the ones that responded to multiple song stimuli, exhibited biased responses to one particular song (Extended Data Fig. 2-1*B*). Therefore, we further investigated the response selectivity, especially regarding FS, by calculating the *d*′ values of RS between two song stimuli. Almost half of responsive BS neurons (51.9%, 107/206) showed selective responses with *d*′ > 0.5 compared with all possible pairs with other stimuli (Fig. 2*I*, right). The proportion of FS-selective neurons was similar to that of the neurons selective for other songs (*χ*^2^_(9)_ = 21.33; *p* = 0.011, *χ*^2^ test; all *p* > 0.05, post hoc Fisher's exact tests; Fig. 2*I*, right), unlike previous reports in male juveniles, in which a larger fraction of neurons showed selective responses to experienced TS (Yanagihara and Yazaki-Sugiyama, 2016; Katic et al., 2022). NS neurons responded to a significantly greater number of song stimuli than BS neurons (NS, 5.8 ± 2.8 stimuli; BS, 2.5 ± 1.7 stimuli; *U* = 11,860; *p* = 2.02 × 10^−17^; *r* = 0.50, Mann–Whitney *U* test; Fig. 2*F*, top, *G*, left; Extended Data Fig. 2-1*C*). Despite most NS neurons responding to multiple songs, more than a half of responsive NS neurons (59.4%, 41/69) showed selective (biased) responses to one song (Fig. 2*I*, left). We found most selective NS neurons were selective to female calls, and the proportion of female-call-selective NS neurons was significantly higher than that of the neurons selective for other songs (*χ*^2^_(9)_ = 94.10; *p* = 2.44 × 10^−16^, *χ*^2^ test; OR = 0.493; all *p* < 0.05 except for FC vs ZF4, post hoc Fisher's exact tests; Fig. 2*I*, left), whereas the proportion of FS-selective NS neurons was not higher than that of neurons selective for other songs (all *p* > 0.05, post hoc Fisher's exact tests). These findings suggest that NCM neuronal ensembles are responsive to a variety of zebra finch vocalizations, and no neuronal memory traits of FS remain in the NCM as selective neurons unlike in NCM of male juveniles. However, we cannot preclude the possibility that FS-selective neurons are located more lateral or rostral part of NCM where our recording did not cover (Extended Data Fig. 2-1*A*).

10.1523/ENEURO.0164-26.2026.f2-1 Figure 2-1 NCM neuronal responses to the song stimulus series. A: The estimated recording tracks confirmed by the traces of electrodes in post-hoc histological analysis in each bird. Red and blue dashed lines denote the tracks of electrode in normally reared and isolated birds, respectively. B & C: Sound spectrograms of each song in the song stimulus series (top) and raster plots of neuronal responses to those stimuli of representative NCM BS (A) and NS (B) neurons (bottom). Pink shaded areas in raster plots show the time window of stimuli playback. Download Figure 2-1, TIF file.

### NCM BS neurons of adult females were not biased to the FS element

As we found no greater proportions of FS-selective neurons in both BS and NS neurons, we further examined whether NCM neurons responded to elements of FS, especially BS neurons which respond only to a small part of songs, by probing NCM neurons from another subset of adult females with the song element stimulus series (NS, 21 neurons, 1–7 neurons/bird; BS, 205 neurons, 16–45 neurons/bird; six birds), which comprised 10 song elements for each of eight element type (totally 80 elements), as reported in our previous study (Cheng and Yazaki-Sugiyama, 2025; Fig. 3*A*). The majority of NCM neurons (NS, 100.0%, 21/21; BS, 63.9%, 131/205) responded significantly to at least one song element in the stimulus series. As found in adult males (Cheng and Yazaki-Sugiyama, 2025), almost half of responsive BS neurons showed selective responses to one element type, exhibiting *d*′ > 0.5 in the average RS to the elements in the same type in comparison with all the other element types (51.1%, 67/131; Fig. 3*B*,*C*). The proportions of BS neurons selective for each element type were similar except to those selective for Types 4 (3.0%, 2/67) and 5 (3.0%, 2/67), which were significantly less than those selective for Type 3 (22.4%, 15/67; *χ*^2^_(7)_ = 22.43; *p* = 0.002, *χ*^2^ test; OR = 0.65; *p* = 0.048; *ϕ* = 0.05, post hoc Fisher's exact tests; Fig. 3*C*). These proportions contrasted with adult males in which most of type-selective BS neurons were selective for Type 6 element which has female distance-call acoustical structures. As we failed to find a distinct proportion of neurons selective to FS, we further examined if NCM BS neurons in each female were selective to specific element types included in FS (Fig. 3*D*,*E*). We found that the proportions of neurons that were selective to the element types in FS in one bird were not different from those selective to the element types in UF (FS, 64.0 ± 28.4%; UF, 46.7 ± 31.3%; *W* = 1.0; *p* = 0.25; *r* = 0.81, Wilcoxon signed-rank test). Notably, nonselective BS neurons responded to significantly smaller numbers of song elements in the stimuli than selective BS neurons (nonselective, 7.9 ± 13.8 elements; selective, 8.7 ± 9.0 elements; *U* = 1,588; *p* = 0.01; *r* = 0.22, Mann–Whitney *U* test), whereas nonselective BS neurons responded to a comparable number of element types as selective BS neurons (nonselective, 3.0 ± 2.1 element types; selective, 3.2 ± 2.0 element types, *U* = 1,979; *p* = 0.438; *r* = 0.07, Mann–Whitney *U* test; Fig. 3*B*), suggesting that nonselective BS neurons were selective for specific elements, not element types as found in adult males (Cheng and Yazaki-Sugiyama, 2025). We further examined if these nonselective neurons responded particularly to the elements similar to the FS elements (Fig. 4). The element similarity measured with Sound Analysis Pro 2011 was not perfectly corresponding to element types. The proportions of elements that were similar to the FS elements were comparable to those of elements similar to the UF elements within the element to which nonselective neurons responded in each bird (all *p* > 0.05, Wilcoxon signed-rank tests; Fig. 4). NS neurons responded to significantly greater numbers of song elements and element types than BS neurons (NS, 51.5 ± 18.0 elements; BS, 8.3 ± 11.6 elements; *U* = 2,646; *p* = 9.69 × 10^−12^; *r* = 0.55, Mann–Whitney *U* test; NS, 7.1 ± 1.4 element types; BS, 3.1 ± 2.1 element types; *U* = 2,539; *p* = 2.99 × 10^−10^; *r* = 0.50, Mann–Whitney *U* test; Extended Data Fig. 3-1*A*). More than half of element-responsive NS neurons (61.9%, 13/21) exhibited selective (biased) responses to one element type, most of which (8/13) were to Type 6 which has female distance-call acoustic structures (Extended Data Fig. 3-1*B*). Those suggest memories of FS elements are not stored in NCM neurons, at least not as neurons showing selective responsiveness.

10.1523/ENEURO.0164-26.2026.f3-1 Figure 3-1 NS neuronal responses to the song element stimulus series A: Heatmaps of the response strength (z-score) of type-selective (top) and nonselective (bottom) NS neurons to 80 song elements in the song element stimulus series in normally reared females. B: Proportions of type-selective NS neurons to each element type in normally reared females (n = 21 neurons) (Type 1, 9.5%; Type 2, 9.5%; Type 3, 0.0%; Type 4, 0.0%; Type 5, 0.0%; Type 6, 38.1%; Type 7, 0.0%; Type 8, 4.8%). C: Heatmaps of the response strength (z-score) of type-selective (top) and nonselective (bottom) NS neurons to 80 song elements in the song element stimulus series in isolated females. D: Proportions of type-selective NS neurons to each element type in isolated females (n = 13 neurons) (Type 1, 7.7%; Type 2, 15.4%; Type 3, 0.0%; Type 4, 0.0%; Type 5, 0.0%; Type 6, 30.8%; Type 7, 7.7%; Type 8, 0.0%). Download Figure 3-1, TIF file.

### Developmental song experiences shape NCM neuronal response properties to songs, but not to song elements

We did not find selectivity to the FS or FS elements in the responsiveness of NCM neurons, whereas previous studies showed that the neurophysiological properties of NCM neurons in female adults are shaped by developmental auditory experiences (Lauay et al., 2005; Terpstra et al., 2006; Kudo et al., 2020; Moseley and Meliza, 2024). Also, while the proportions of FS-selective neurons were not different from proportions of selective neurons to other songs, we hypothesized they may be greater than the proportions of FS-selective neurons in the females that have no FS experiences. We then examined whether the response properties of NCM neurons to songs and their elements were shaped by FS experiences. We recorded from NCM neurons in the adult females that were isolated from their father starting at 10 dph and reared in a sound attenuation chamber (NS, 21 neurons, 1–10 neurons/bird; BS, 217 neurons, 17–93 neurons/bird; four isolated birds). Those isolated female adults showed normal social behavior with their mother and female siblings. The females with no FS experiences are reported to show no preference toward FS over UF (Clayton, 1988; Riebel, 2000, 2003). The spike width of NS and FRs of BS neurons in isolated adult females were comparable to those of NS and BS neurons in the normally reared birds [NS trough-to-peak duration, (isolated) 0.21 ± 0.04 ms (normally reared) 0.20 ± 0.04 ms; *U* = 700; *p* = 0.243; *r* = 0.12, Mann–Whitney *U* test; BS FRs, (isolates) 0.32 ± 0.49 spikes/s (normally reared) 0.32 ± 0.84 spikes/s; *U* = 29,836; *p* = 0.459; *r* = 0.03, Mann–Whitney *U* test], while the spike widths of BS neurons in isolated birds were shorter than those of BS neurons in normally reared birds [(isolated) 0.41 ± 0.05 ms (normally reared) 0.43 ± 0.06 ms; *U* = 38,262; *p* = 7.52 × 10^−6^; *r* = 0.20, Mann–Whitney *U* test], and the FRs of NS neurons in isolated birds were higher than those of NS neurons in normally reared birds [(isolated) 5.72 ± 3.15 spikes/s (normally reared) 5.36 ± 6.94 spikes/s; *U* = 603; *p* = 0.048; *r* = 0.20, Mann–Whitney *U* test; Fig. 5*A*–*C*). Comparable proportions of NS and BS NCM neurons in the isolated females responded significantly to at least one song in the song stimulus series as observed in normally reared birds (NS, 95.2%, 20 /21; BS, 83.9%, 182/217). Consistent with the finding in the normally reared birds, NS neurons responded to almost all stimuli, whereas BS neurons in the isolated birds responded to only a small subset of stimuli in the song stimulus series (NS, 8.0 ± 2.3 stimuli; BS, 3.3 ± 2.4 stimuli; *U* = 3,288; *p* = 1.92 × 10^−9^; *r* = 0.42, Mann–Whitney *U* test; Fig. 5*D*,*E*). Notably, both NS and BS neurons in isolated birds responded to a significantly greater number of stimuli within the song stimulus series than those of normally reared birds [NS, (isolated) 8.0 ± 2.3 stimuli (normally reared) 5.8 ± 2.8 stimuli; *U* = 1,006; *p* = 0.002; *r* = 0.33, Mann–Whitney *U* test; BS, (isolated) 3.3 ± 2.4 stimuli (normally reared) 2.5 ± 1.7 stimuli; *U* = 21,891; *p* = 0.003; *r* = 0.15, Mann–Whitney *U* test; Figs. 5*E*, 2*G*]. Each song stimulus was responded by different combinations of BS neurons in individual isolated birds (Fig. 5*D*, bottom, *F*, right). The proportions of selective BS neurons in one isolated bird were significantly higher than those in one normally reared bird [(isolated) 69.4 ± 5.4% (normally reared) 53.1 ± 8.1%; *U* = 27; *p* = 0.012; *r* = 0.74, Mann–Whitney *U* test; Figs. 5*G*, right, 2*I*, right], a stark contrast to the previous report in isolated male juveniles in which only a small number of selective neurons were found (Yanagihara and Yazaki-Sugiyama, 2016). The proportion of BS neurons selective to FS, to which the isolated birds had not been exposed after 10 dph, was not different from that in normally reared birds [(isolated) 5.5 ± 3.2% (normally reared) 6.5 ± 3.0%; *U* = 16.5; *p* = 0.705; *r* = 0.14, Mann–Whitney *U* test]. In addition, the proportion of FS-selective neurons was not different from that of neurons selective to any other songs (*χ*^2^_(9)_ = 21.86; *p* = 0.009; *χ*^2^ test, all *p* > 0.05, post hoc Fisher's exact tests; Fig. 5*G*, right). These data suggest that developmental song experiences shape NCM response properties to songs in general, but not specifically selective responsiveness to FS.

Finally, we examined NCM neuronal responsiveness to song elements in isolated female adults by probing with the element stimulus series (NS, 13 neurons, 1–5 neurons/bird; BS, 157 neurons, 22–42 neurons/bird; five isolated birds). The majority of NCM neurons in isolated birds (NS, 100.0%, 13/13; BS, 70.1%, 110/157) exhibited significant responses to at least one song element. As observed in normally reared birds, NS neurons responded to most of the song elements (50.2 ± 18.1 elements) and element types (7.2 ± 1.9 element types), yet the majority of them (61.5%, 8/13) exhibited selective responses to Type 6 (Extended Data Fig. 3-1*C*,*D*). We also found that almost half of BS neurons (49.1%, 54/110) in isolated birds showed selective responses to one element type, which was comparable to the proportion observed in the normally reared birds (Figs. 6*A*, *B*, 3*B*,*C*). The proportions of selective neurons to each element type were similar to each other except significantly fewer neurons were selective for Type 5 (1.9%, 1/54) than that to Type 6 (27.8%, 15/54; *χ*^2^_(7)_ = 21.39; *p* = 0.003, *χ*^2^ test; OR = 1.26; *p* = 0.045; *ϕ* = 0.02, post hoc Fisher's exact test; Fig. 6*A*,*B*), as seen in the normally reared females. Unlike the responsiveness to song stimuli, the number of elements and element types BS neurons responded to in isolated females was comparable to those observed in normally reared females [elements, (isolated) 7.3 ± 3.0 elements (normally reared) 8.3 ± 3.1 elements; *U* = 6,845; *p* = 0.501; *r* = 0.04, Mann–Whitney *U* test; element types, (isolated) 3.0 ± 2.2 types (normally reared) 3.1 ± 2.1 types; *U* = 6,840; *p* = 0.488; *r* = 0.04, Mann–Whitney *U* test; Figs. 6*A*, 3*B*]. We further found that the proportions of neurons that were selective to the element types in the genetic FS of each isolated bird were similar to those selective to the element types in UF (FS, 52.9 ± 21.8%; UF, 52.9 ± 21.8%; *W* = 1.0; *p* = 1.0; *r* = 0.91, Wilcoxon signed-rank test; Fig. 6*C*,*D*). Consistent with the findings in normally reared birds, the comparative proportions of the elements to which nonselective neurons responded were similar to the elements of their genetic FS and to the elements of UF in three out of five isolated birds (*p* > 0.05, Wilcoxon signed-rank tests). In another two isolated birds nonselective neurons responded to more elements that were similar to UF elements than those similar to FS elements [Bird 1, (UF) 48.2 ± 41.3% (FS) 32.5 ± 30.4%; *W* = 0.0; *p* = 0.031; *r* = 0.89; Bird 2, (UF) 43.2 ± 24.2% (FS) 2.5 ± 5.5%; *W* = 0.0; *p* = 0.008; *r* = 0.88, Wilcoxon signed-rank tests; Fig. 7]. Taken together, our results suggest that female NCM neurons detect zebra finch song elements independent of developmental song experiences while auditory exposure to song shapes properties of their auditory responses to songs which comprise combinations of elements, but not specifically to FS.

### Decrease FS discriminability with reduced contributions of FS-responsive neurons

While we did not find memory traits of FS as neuronal selective responsiveness to FS or its elements, ablating FS-responsive neurons decreased the number of hops selectively triggering FS playback. To understand how ablating FS-responsive neurons reduces preference behavior to FS, we recorded neurons before and after the induction of cell death in another subset of adult females (Extended Data Fig. 1-2*A*). As the number of hops toward both FS and UF substantially decreased by implanting the electrode with AAV injections (Before, 135 ± 108 hops; After, 41 ± 17 hops), assessing song preference in the females, in which NCM neurons were recorded for song responsiveness, was not possible. Although recording the auditory responsiveness from the neurons in the brain area subjected to surgery was challenging, we successfully recorded NCM neuronal responses before and after neuronal ablation from two birds [Bird 1, (preablation) 19 neurons, (postablation) 32 neurons; Bird 2, (preablation) 29 neurons, (postablation) 27 neurons]. The proportion of FS-responsive BS neurons decreased after the cell death induction in FS-responsive neurons as expected (Extended Data Fig. 1-2*B*, left, *C*, left), while the proportion of responsive BS neurons within recorded neurons was relatively smaller compared with the previous recording in the normally reared birds without AAV injections (preablation, 19/40 BS neurons; postablation, 23/43 BS neurons; normally reared birds, 206/286 BS neurons). In contrast, the proportion of neurons responding to FS did not change before and after DOX administration with no song playback (Extended Data Fig. 1-2*B*, right, *C*, right). This strongly suggests the reduction of the proportion of neurons responsive to FS by inducing neuronal cell death with the DOX administration and FS playback occurred as expected. We then finally tested whether song discriminability to FS differs with a smaller proportion of model neurons that are responsive to FS using decoder-based song prediction by using the RS of BS neurons recorded in the normally reared birds (Figs. 2, 8*A*). The decoding accuracy for FS did not change with the removal of a quarter of model neurons responsive to FS; however, the accuracy reduced when 75 and 90% of model neurons responsive to FS (17 and 20 percent of total song-responsive neurons) were removed from the neuronal ensembles, even in the ensembles including 150 model neurons (Fig. 8*A*,*B*). In contrast, the decoding accuracy for other song stimuli did not change by the reduction of the proportion of model neurons responsive to FS (Fig. 8*C*). Those suggest impaired discriminability for FS in NCM with reduced number of neurons responsive to FS is one possible reason for reduced preference for FS.

## Discussion

In this study, we found that inducing cell death in FS-responsive NCM neurons decreased behavioral preference for FS in female adult zebra finches. Intriguingly, the auditory responsiveness of NCM neurons to zebra finch songs differed among individuals based on developmental song experience, whereas responses to individual song elements did not. In line with the previous studies showing that regional NCM lesioning disrupted preference and individual recognition for specific male songs (Tomaszycki and Blaine, 2014; Lawley et al., 2022; Yu et al., 2023), inducing cell death in the FS-responsive neurons decreased the number of hops triggering FS playback in female zebra finches. Admittedly, identifying the proportions and areas of ablated neurons with virus infections was not possible and cell death was induced in the FS-responsive neurons, including the ones responded to FS both selectively and nonselectively. We found individual variation in the effect of neuronal ablation while the sample size was small. It is impossible to identify which step of song preference behavior, song recognition or preference, was affected by neuronal ablation. We found the proportions of FS-selective neurons were not different between the females with and without developmental experience of FS. Proportions of NCM neurons showing selective responses to the element types included in FS were not greater than those selective to the element types in UF. These results suggest decreasing the number of hops for FS after ablation of FS-responsive neurons is attributed to disrupted FS recognition with decreased number of FS-responsive neurons. This idea is supported by the observation that decoding accuracy for FS reduced with the neuronal ensembles containing smaller proportions of FS-responsive model neurons.

Various behavioral studies have shown that auditory experiences alter the preference of adult females to experienced songs (Clayton, 1987, 1988, 1990; Riebel, 2000; Lauay et al., 2004; Chen et al., 2017; Wang et al., 2022). A recent study also showed that a greater number of NCM neurons expressed pS6 in females exposed to their mate's songs than in those exposed to UF (Catalano and Woolley, 2025). We found that the proportions of selective neurons in isolated adult females were greater than those of normally reared adult females and that the numbers of songs NCM neurons in isolated adult females responded to were greater than those in normally reared adult females, suggesting song experiences alter auditory response properties of NCM neurons to zebra finch songs. Previous studies showed that auditory experiences alter the neurophysiological properties of NCM neurons in both males and females (Kudo et al., 2020; Moseley and Meliza, 2024). Song selectivity or responsiveness of NCM neurons can be altered by the suppression of local GABA inhibitory function in male juveniles (Yanagihara and Yazaki-Sugiyama, 2016; Spool et al., 2021), as also seen in the mammalian cortex (Tremblay et al., 2016; Moore et al., 2018). NS neurons, in which we found a difference in responsiveness to songs between normally reared and isolated females, are suggested to be GABAergic neurons (Spool et al., 2021). In rodents, sensory experiences have been reported to modulate cortical local GABA inhibitory functions (Hensch et al., 1998; Jaio et al., 2006; Griffen and Maffei, 2014). Neuronal circuit modifications with repeated exposure to songs might also modulate song preference, as we found a slight recovery of song preference to FS after 8 weeks of neuronal ablation. Taken together, auditory experiences are suggested to modify auditory responses to a song of NCM neuronal circuits and consequently behavioral song preference.

Interestingly, we found the proportions of song element type-selective neurons and the number of elements and element types the selective neurons responded to in isolated female birds were not different from those in normally reared females. Zebra finch song motifs consist of 3–14 song elements that can be clustered into several types based on their acoustic similarities (Zann, 1993, 1996). Zebra finch song element types are highly conserved across domesticated populations worldwide and in wild-caught individuals in Australia (Lachlan et al., 2016). A fraction of those conserved element types was acoustically similar to some types of calls (e.g., distance calls) and potentially evolved from those calls (Zann, 1993, 1996; Elie and Theunissen, 2016), suggesting no differences in element type selectivity between normally reared and isolated birds might be due to the auditory experience of calls. Zebra finches recognize element/syllable sequence alterations (Chen and ten Cate, 2015; Lawson et al., 2018; Geberzahn and Deregnaucourt, 2020; Ning et al., 2024). Female zebra finches also discriminate mate songs and FS, which are sometimes very similar to their siblings' or their TS (Miller, 1979a; Riebel and Smallegange, 2003; Ihle and Forstmeier, 2013). NCM neurons, especially BS neurons, respond only to specific elements within a song (Schneider and Woolley, 2013; Yanagihara and Yazaki-Sugiyama, 2016; Bottjer et al., 2019; Katic et al., 2022). Recently, we reported that NCM neurons in males are sensitive to the sequences of elements or element types (Cheng and Yazaki-Sugiyama, 2025). Auditory experiences are reported to modify physiological and song-responsive properties of NCM neurons in male and female juveniles (Phan et al., 2006; Yanagihara and Yazaki-Sugiyama, 2016; Kudo et al., 2020; Katic et al., 2022; Moseley and Meliza, 2024). Taken together, our results suggest that NCM neuronal ensembles detect zebra finch syllable elements regardless of song experiences and that song experiences shape local NCM neuronal circuits for detection of song, sequence of elements, and consequently song preference in behavior.

In male juveniles learning to sing, a substantial proportion of NCM neurons refine firing to show selective, almost exclusive responses to a TS after tutor experiences (Yanagihara and Yazaki-Sugiyama, 2016; Katic et al., 2022), and ablating TS-responsive neurons prevents male juveniles from learning TS (Louder et al., 2024). In contrast, we recently reported that the proportion of TS-selective NCM neurons was comparable to that selective for other UF in adult males (Cheng and Yazaki-Sugiyama, 2025). In adult females, NCM neurons showed biased responses to the calls, normally comprised of one or two elements, of their mate or familiar males compared with those of unfamiliar males (Menardy et al., 2012). However, the amount of immediate early gene expressions in NCM is similar between the females exposed to FS and those exposed to UF (Terpstra et al., 2006). Estradiol is reported to modulate song responsiveness particularly in NCM NS neurons in both males and females (Fernandez-Vargas et al., 2024). Blocking estradiol synthesis in NCM does not impair song learning in male juveniles (Vahaba et al., 2020), whereas it alters auditory scene analysis in females (Fernandez-Vargas et al., 2024). Here, we found, in contrast to male juveniles and similar to male adults, the proportion of FS-selective neurons, most of which showed biased, but not exclusive, responses to FS, was comparable to that of neurons selective for other songs in adult females. In addition, the proportion of FS-selective neurons in normally reared females was not different from that in isolated females. These findings suggest that the NCM of female adults does not hold the neuronal substrates of FS memories in the form of selective neurons as in male juveniles. Both juvenile song learning and song recognition in adults are believed to require memory formation of experienced songs. Inactivation of NCM neurons has been reported to inhibit song learning and to disrupt the performance of trained vocal recognition tasks (London and Clayton, 2008; Yu et al., 2023). Those collectively suggest that NCM hold memories of songs for song learning and recognition; however, the way of forming memories and the functions of NCM regarding song memory for juvenile song learning and song recognizing behavior in adults might be different.

We found song-selective and song element-selective neurons in NCM in female adult zebra finches, as has been found in adult males. However, the neurons selective to FS are not suggested to be memory traits like the TS-selective neurons in male juveniles. Rather, NCM is suggested to detect varieties of individual songs as an ensemble. How and where experienced song memories are stored in the brain has yet to be elucidated in adults. Song memories in male juveniles are thought to be used predominantly for song learning. We recently reported that NCM TS-responding neurons in male juveniles transiently project to the song premotor brain region, HVC (Louder et al., 2024). Female zebra finches choose their mate based on their songs, suggesting song memories are used for sexual behavior instead of song learning. The previous study showed infusing dopamine agonist to NCM with song playback increased preference for less-preferred songs of adult females (Barr et al., 2021), and here we show that the NCM is involved in female FS preference behavior as well. Further studies on the downstream brain areas from NCM for each corresponding behavior with cellular-level analysis with interareal neuronal circuit would inform us on the sexually dimorphic neuronal pathway for sex-specific auditory behaviors.

## Synthesis

Reviewing Editor: Luke Remage-Healey, University of Massachusetts Amherst Decisions are customarily a result of the Reviewing Editor and the peer reviewers coming together and discussing their recommendations until a consensus is reached. When revisions are invited, a fact-based synthesis statement explaining their decision and outlining what is needed to prepare a revision will be listed below. The following reviewer(s) agreed to reveal their identity: NONE. Note: If this manuscript was transferred from JNeurosci and a decision was made to accept the manuscript without peer review, a synthesis may not be available.

The authors have been responsive to the prior concerns of the two reviewers from J Neurosci in their revision. As an expert in the field, I have reviewed the manuscript as well as the earlier reports of the two reviewers, and they have addressed the major and minor concerns of both original reviewers.
