# Supplementary figures and images for: Functions of the Auditory Forebrain for Song Memory and Preference Behavior in Female Zebra Finches
Source: eNeuro. 2026 Jun 24;13(6):ENEURO.0164-26.2026. doi: 10.1523/ENEURO.0164-26.2026 (PMC13421842; doi:10.1523/ENEURO.0164-26.2026)

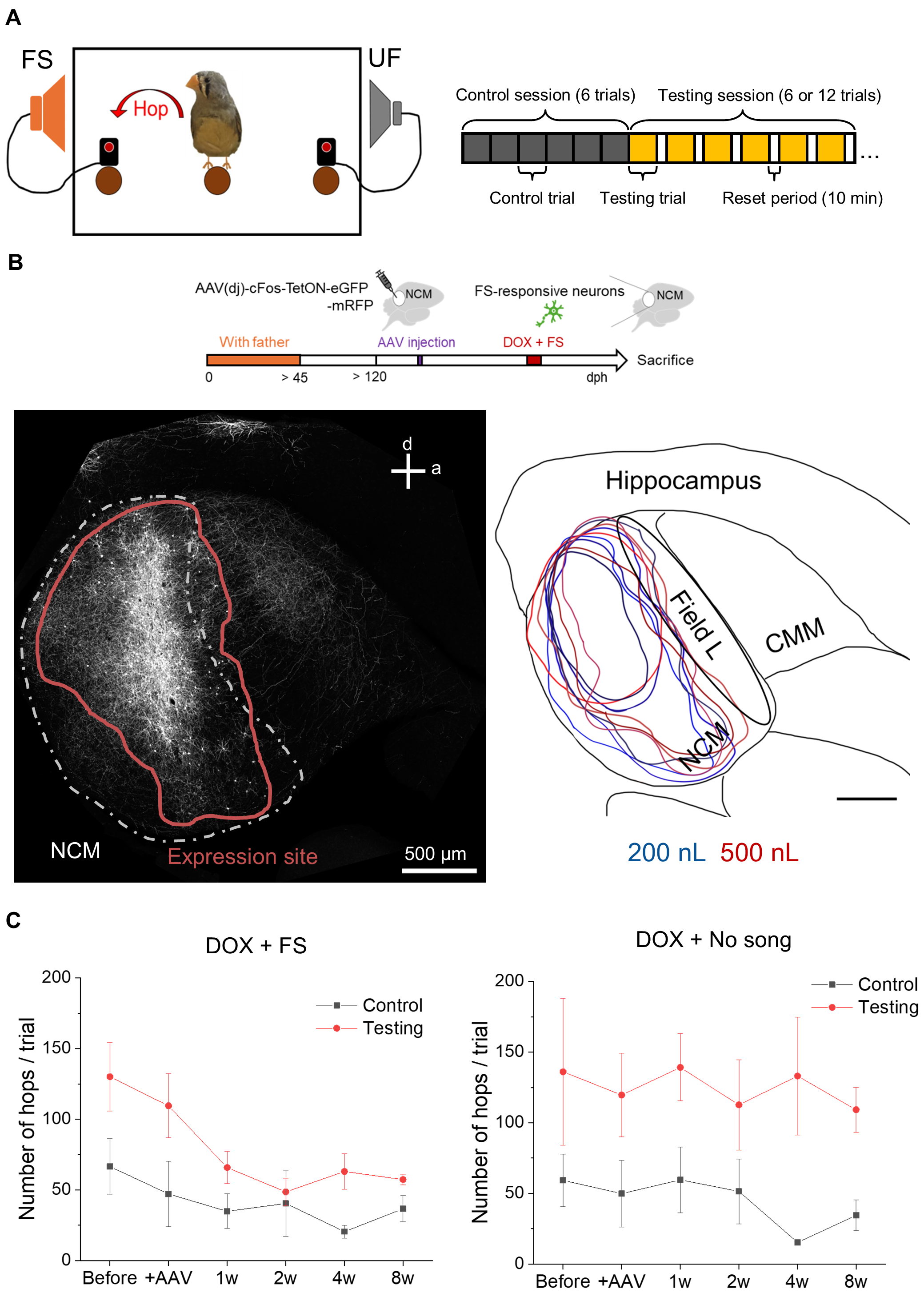

Supplement: Figure 1-1 — Adult female zebra finches consistently hop more during testing trials than during control trials. A: Schematic of experimental perch-hop assay setup used in this study (left) and the diagram of experiment timelines (right). Hopping on the two experimental perches triggered song playbacks during testing sessions, whereas it did not during control sessions. B: The timeline for visualizing FS-responsive neurons (top), a representative image of NCM slice of injection site in the adult female zebra finch injected with AAV(dj)-cFos-TetON-eGFP (bottom left), and a drawing of the brain slice including NCM (bottom right). The colored contours denote the areas the GFP-positive or RFP-positive somata were found in each hemisphere injected with 200 nL (blue) or 500 nL (red) of AAV. a, anterior; d, dorsal. Scale bar, 500 μm. C: Average number of hops across birds performed during control (black) and testing trials (red) before and after the virus injection and NCM neuronal ablation in experimental birds (DOX + FS; N = 6) and control birds (DOX + No song; N = 4). Error bars, standard errors. Download Figure 1-1, TIF file. [file eneuro-13-ENEURO.0164-26.2026-s001.tif]

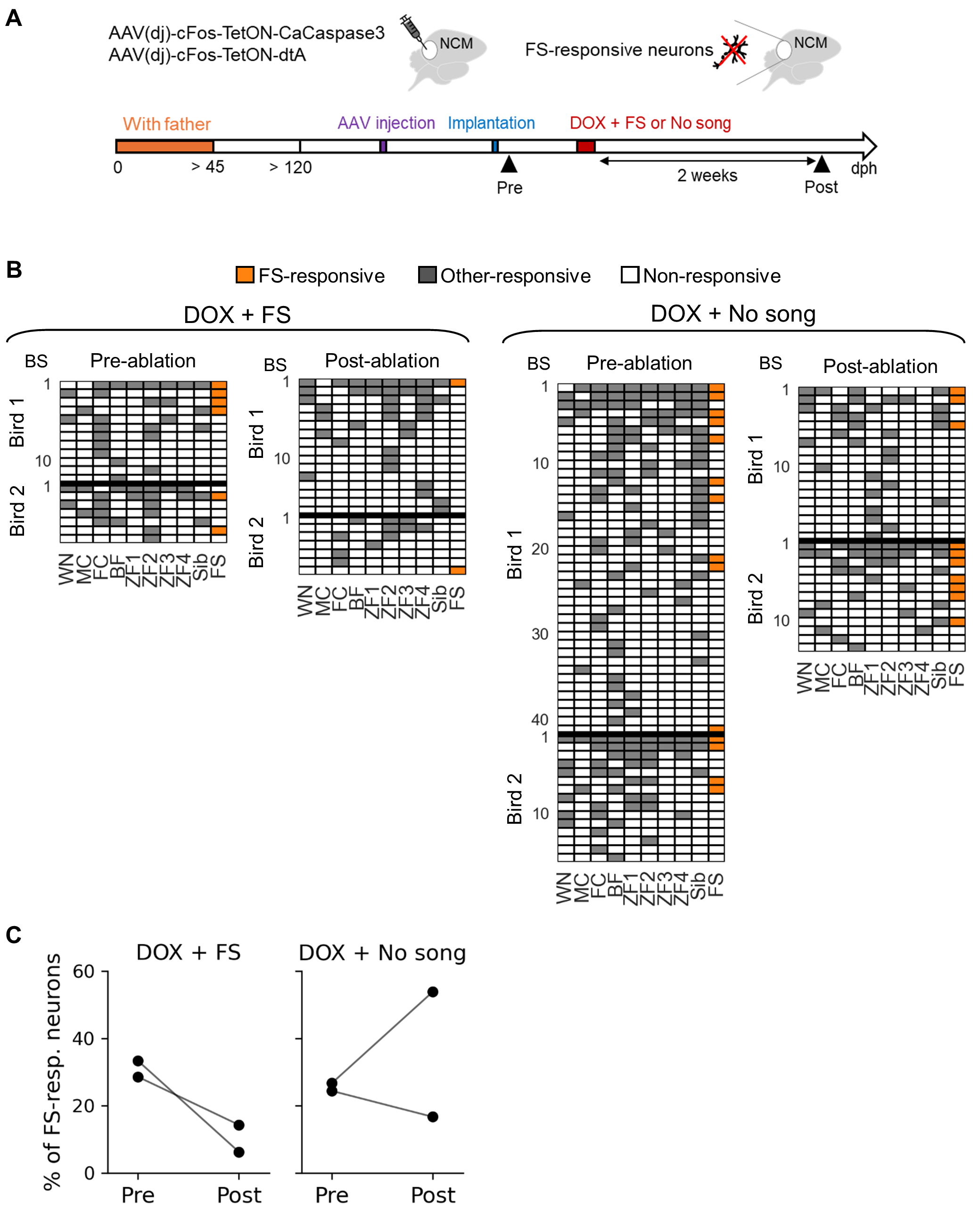

Supplement: Figure 1-2 — Proportion of FS-responsive neurons decreased after ablating FS-responsive neurons. A: Timeline of extracellular recordings before and after the neuronal ablation. Arrowheads indicate the time points of extracellular recordings. B: Responses of individual BS neurons to each song stimulus recorded from individual experimental (DOX + FS) and control female birds (DOX + No song) before (Pre-ablation) and after (Post-ablation) neuronal ablation. Filled boxes denote a significant response (p < 0.05, Wilcoxon signed-rank test), among which orange boxes denote responses to FS. C: Proportion of FS-responsive BS neurons within all responsive BS neurons before (Pre) and after (Post) neuronal ablation in single experimental (DOX + FS) and control (DOX + No song) females. The proportions of FS-responsive neurons decreased after neuronal ablation in experimental birds, but not in control birds. Download Figure 1-2, TIF file. [file eneuro-13-ENEURO.0164-26.2026-s002.tif]

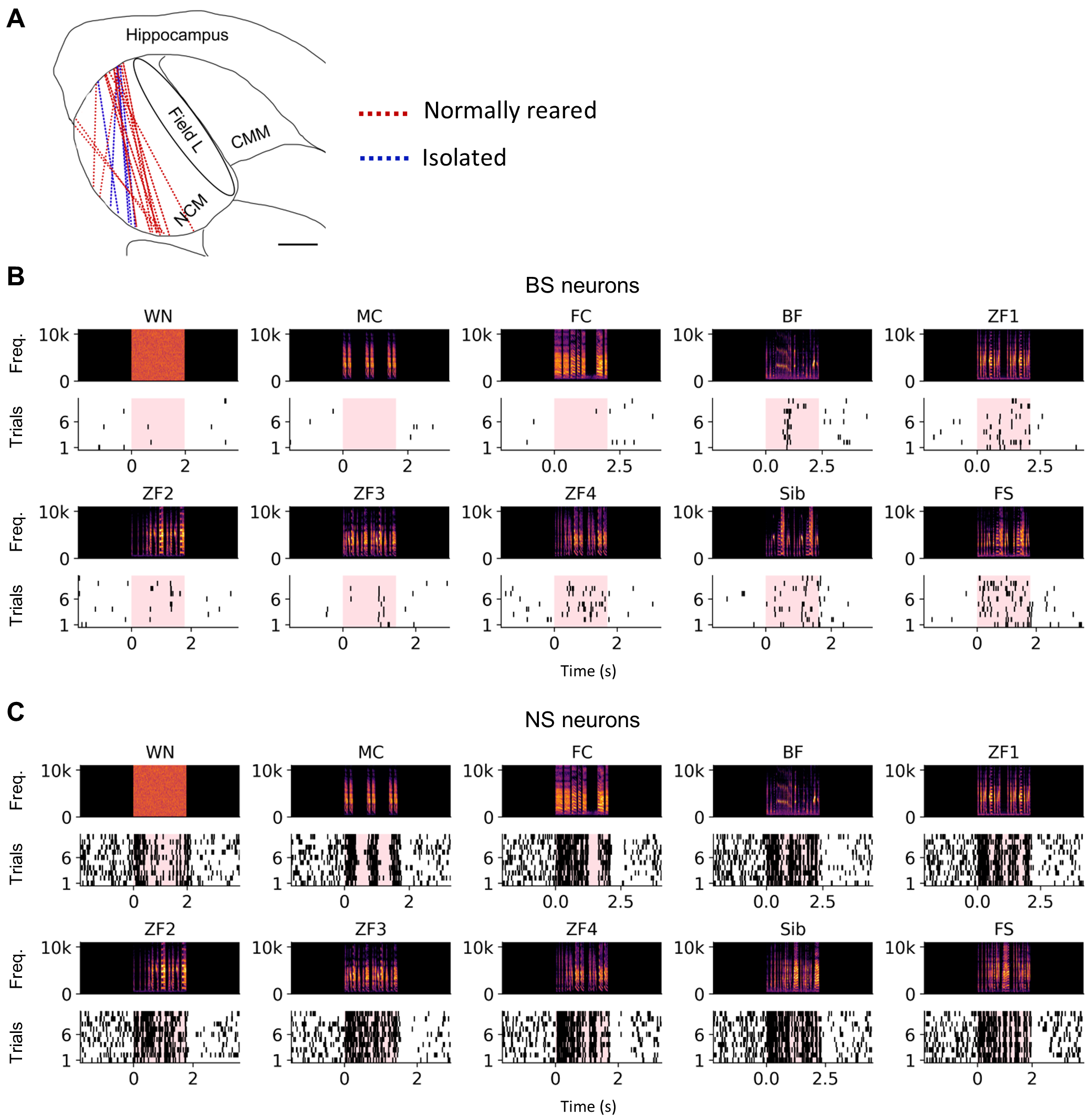

Supplement: Figure 2-1 — NCM neuronal responses to the song stimulus series. A: The estimated recording tracks confirmed by the traces of electrodes in post-hoc histological analysis in each bird. Red and blue dashed lines denote the tracks of electrode in normally reared and isolated birds, respectively. B & C: Sound spectrograms of each song in the song stimulus series (top) and raster plots of neuronal responses to those stimuli of representative NCM BS (A) and NS (B) neurons (bottom). Pink shaded areas in raster plots show the time window of stimuli playback. Download Figure 2-1, TIF file. [file eneuro-13-ENEURO.0164-26.2026-s004.tif]

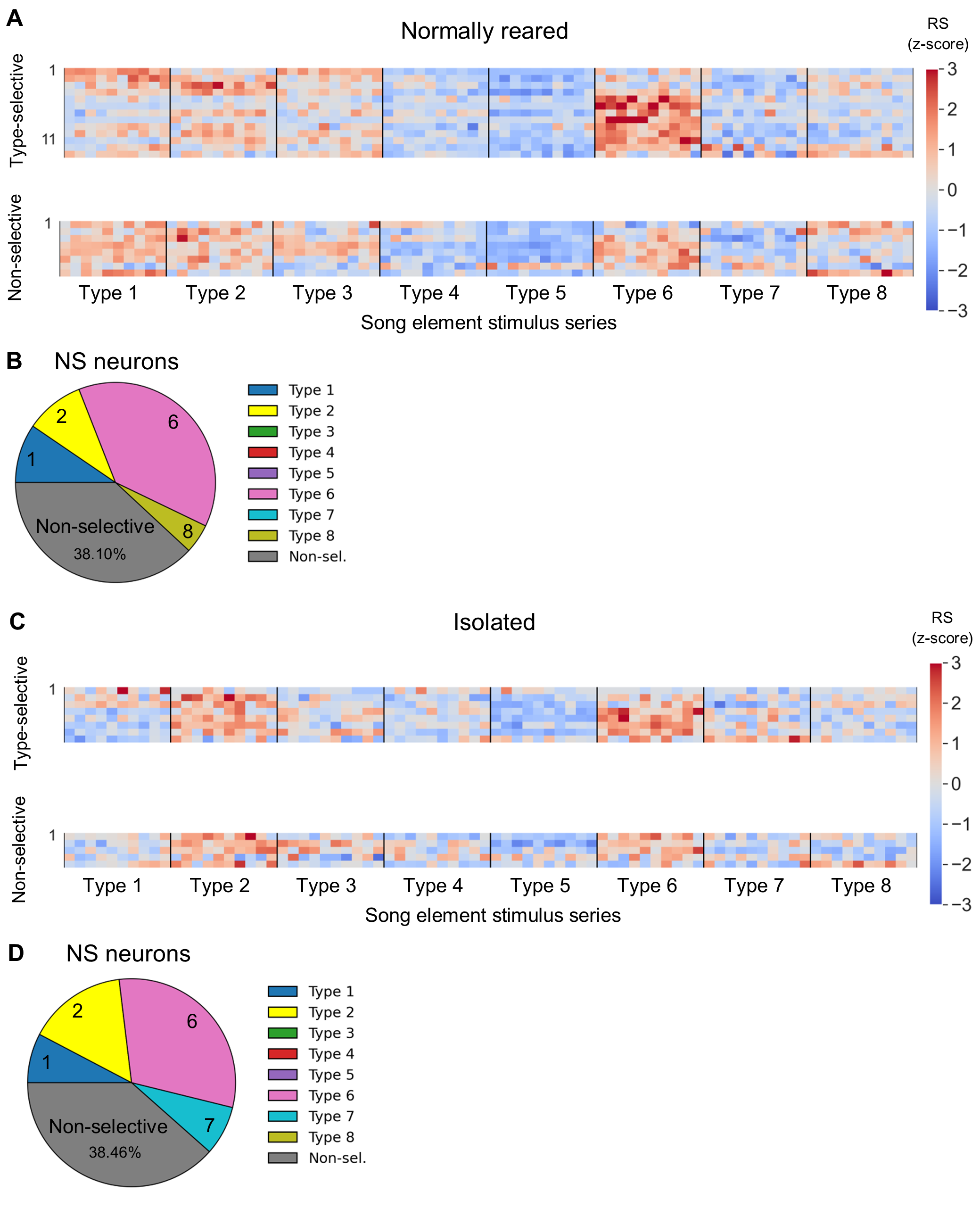

Supplement: Figure 3-1 — NS neuronal responses to the song element stimulus series A: Heatmaps of the response strength (z-score) of type-selective (top) and nonselective (bottom) NS neurons to 80 song elements in the song element stimulus series in normally reared females. B: Proportions of type-selective NS neurons to each element type in normally reared females (n = 21 neurons) (Type 1, 9.5%; Type 2, 9.5%; Type 3, 0.0%; Type 4, 0.0%; Type 5, 0.0%; Type 6, 38.1%; Type 7, 0.0%; Type 8, 4.8%). C: Heatmaps of the response strength (z-score) of type-selective (top) and nonselective (bottom) NS neurons to 80 song elements in the song element stimulus series in isolated females. D: Proportions of type-selective NS neurons to each element type in isolated females (n = 13 neurons) (Type 1, 7.7%; Type 2, 15.4%; Type 3, 0.0%; Type 4, 0.0%; Type 5, 0.0%; Type 6, 30.8%; Type 7, 7.7%; Type 8, 0.0%). Download Figure 3-1, TIF file. [file eneuro-13-ENEURO.0164-26.2026-s005.tif]
