# Supplementary material for: Functions of the Auditory Forebrain for Song Memory and Preference Behavior in Female Zebra Finches
Source: eNeuro. 2026 Jun 24;13(6):ENEURO.0164-26.2026. doi: 10.1523/ENEURO.0164-26.2026 (PMC13421842; doi:10.1523/ENEURO.0164-26.2026)
Supplement: Figure 1-3 — The list of female zebra finches used in this study showing the ages removed from their breeding cages, subjected to the first preference test, and subjected to AAV injections or electrophysiological recordings. Download Figure 1-3, DOCX file. [file eneuro-13-ENEURO.0164-26.2026-s003.docx]

**Figure 1-3:** The list of female zebra finches used in this study showing the ages removed from their breeding cages, subjected to the first preference test, and subjected to AAV injections or electrophysiological recordings.

| Bird ID | Conditions | NCM | Experiments | Cage removal age (dph) | Preference test age (dph) | Experiment age (dph) |
| --- | --- | --- | --- | --- | --- | --- |
| G710 | Normal | Bilateral | AAV-exp | 77 | 164 | 171 |
| Y721 | Normal | Bilateral | AAV-exp | 83 | 122 | 129 |
| B705 | Normal | Bilateral | AAV-exp | 56 | 182 | 185 |
| B711 | Normal | Bilateral | AAV-exp | 83 | 188 | 192 |
| O716 | Normal | Bilateral | AAV-exp | 73 | 124 | 135 |
| G736 | Normal | Bilateral | AAV-exp | 91 | 127 | 142 |
| Y748 | Normal | Bilateral | AAV-ctrl | 75 | 129 | 140 |
| Y746 | Normal | Bilateral | AAV-ctrl | 75 | 178 | 188 |
| Y745 | Normal | Bilateral | AAV-ctrl | 73 | 151 | 151 |
| O757 | Normal | Bilateral | AAV-ctrl | 108 | 136 | 137 |
| O787 | Normal | Left | ephys-song | 72 | 128 | 200 |
| Y788 | Normal | Right | ephys-song | 122 | 166 | 181 |
| B780 | Normal | Left | ephys-song | 62 | 249 | 261 |
| O798 | Normal | Right | ephys-song | 65 | 146 | 147 |
| B759 | Normal | Right | ephys-song | 84 | 135 | 518 |
| G806 | Normal | Left | ephys-song | 90 | 148 | 202 |
| O766 | Normal | Left | ephys-song | 79 | 502 | 635 |
| O839 | Normal | Right | ephys-element | 62 | 190 | 217 |
| R831 | Normal | Left | ephys-element | 52 | 277 | 286 |
| R837 | Normal | Right | ephys-element | 62 | 234 | 247 |
| G849 | Normal | Left | ephys-element | 45 | 179 | 199 |
| B836 | Normal | Right | ephys-element | 66 | 281 | 283 |
| G856 | Normal | Left | ephys-element | 45 | 140 | 143 |
| B804 | Isolated | Right | ephys-song | 10 | NA | 131 |
| G809 | Isolated | Right | ephys-song | 10 | NA | 123 |
| G853 | Isolated | Right | ephys-both | 10 | NA | 139 |
| G855 | Isolated | Right | ephys-both | 10 | NA | 201 |
| R849 | Isolated | Left | ephys-element | 10 | NA | 124 |
| Y856 | Isolated | Left | ephys-element | 10 | NA | 169 |
| Y865 | Isolated | Left | ephys-element | 10 | NA | 129 |
